# DEAD-box RNA helicase 21 interacts with the nucleocapsid protein and inhibits porcine deltacoronavirus infection through upregulation of interferon responses

**DOI:** 10.1128/jvi.01169-26

**Published:** 2026-09-30

**Authors:** Nannan Zhang, Yanyan Xu, Yanying Chen, Mingyue Ma, Wenjie Lai, Shengwei Zhong, Gaofeng Cai, Ting Wang, Bin Li, Qi Peng, Hui Jiang

**Affiliations:** 1 College of Bioscience and Engineering, Jiangxi Agricultural University 91595, Nanchang, China; 2 Institute of Pathogenic Microorganism, Jiangxi Agricultural University 91595, Nanchang, China; 3 Jiangxi Provincial Key Laboratory for Animal Health, College of Animal Science and Technology, Jiangxi Agricultural University 91595, Nanchang, China; 4 Key Laboratory of Veterinary Biological Engineering and Technology, Institute of Veterinary Medicine, Jiangsu Academy of Agricultural Sciences 117941, Nanjing, China; 5 Jiangsu Co-innovation Center for Prevention and Control of Important Animal Infectious Diseases and Zoonoses, Yangzhou University 38043, Yangzhou, China; 6 College of Animal Science and Technology, Jiangxi Agricultural University 593244, Nanchang, China; University of Michigan Medical School, Ann Arbor, Michigan, USA

**Keywords:** porcine deltacoronavirus, DEAD-box RNA helicase 21, nucleocapsid protein, interferon response, viral replication

## Abstract

DEAD-box RNA helicase 21 (DDX21), a conserved RNA helicase of the DEAD-box family, serves as a key regulator of multiple fundamental cellular processes, including RNA metabolism, ribosome biogenesis, transcriptional regulation, and genomic stability. Our previous work has demonstrated an interaction between DDX21 and the nucleocapsid (N) protein of porcine deltacoronavirus (PDCoV). However, the specific role of DDX21 during PDCoV infection remains largely elusive. In the present study, we show that overexpression of DDX21 significantly restricts PDCoV infection in PK15, IPEC-J2, and HeLa cells, whereas knockout of DDX21 markedly enhances PDCoV replication in HeLa cells. Mechanistically, we identified that the N-terminal domainNTD of PDCoV N binds to the 1–217 amino acid region of DDX21, thereby inducing the cytoplasmic redistribution of DDX21 from the nucleus. Furthermore, DDX21-mediated suppression of PDCoV replication coincides with elevated interferon-β (IFN-β) production and interferon-stimulated geneISG expression. Importantly, PDCoV infection significantly downregulates DDX21 expression to antagonize its antiviral function, and similar antagonistic effects are observed for the coronaviruses from different genera. Collectively, our findings demonstrate that DDX21 interacts with the nucleocapsid protein and correlates with altered interferon signaling during coronavirus infection, which provides novel insights into the development of strategies for the prevention and control of coronavirus infections.

**IMPORTANCE:**

Porcine deltacoronavirus (PDCoV) is an emerging swine coronavirus that infects pigs of all ages and causes substantial economic losses to the global swine industry. DEAD-box RNA helicase 21 (DDX21) acts as a key regulator of multiple fundamental cellular processes. However, its regulatory mechanism underlying PDCoV infection remains largely unclear. In this study, we found that DDX21 significantly inhibits PDCoV replication *in vitro*. Conversely, PDCoV infection markedly downregulates DDX21 expression. Furthermore, we demonstrated that the N-terminal 1–217 amino acid domain of DDX21 specifically interacts with the N-terminal domain (NTD) of the PDCoV nucleocapsid (N) protein. This interaction facilitates the nuclear-to-cytoplasmic translocation of DDX21, which correlates with an elevated host immune response. The upregulated interferon signature coincides with suppressed PDCoV replication. In addition, DDX21 exhibits antiviral activity against multiple coronaviruses from different genera. Collectively, this study provides novel insights into host-coronavirus interaction mechanisms and identifies a candidate target for anti-CoV therapeutic development.

## INTRODUCTION

Coronaviruses (CoVs) constitute a large family of enveloped, positive-sense single-stranded RNA viruses that are classified into four genera (*Alpha, Beta, Gamma*, and *Delta*) and pose a persistent and severe threat to global human and animal health (1). With the largest known RNA viral genomes (25–32 kilobases), CoVs exhibit distinct genetic characteristics across genera: *Alpha*- and *Beta*-CoVs encode 16 nonstructural proteins (nsp1–nsp16), while *Gamma*- and *Delta*-CoVs lack the N-terminal nsp1 and encode 15 nsps (nsp2–nsp16) (2–4). Among the structural proteins of CoVs, the nucleocapsid (N) protein is a multifunctional core factor that mediates viral genome packaging, replication, and virion assembly (5–7). More importantly, the N protein of CoVs orchestrates intricate viral-host interactions to subvert the host innate immune response, a critical step in viral immune evasion and successful infection of host cells (8–10). Porcine deltacoronavirus (PDCoV) causes acute severe diarrhea, vomiting, and high mortality in neonatal piglets, leading to considerable economic losses in the global swine industry (11, 12). To date, the molecular mechanisms underlying the host innate immune defense against CoVs and the viral counterstrategies remain incompletely elucidated, hindering the development of effective prevention and control measures for CoV infections.

The host innate immune system serves as the principal frontline barrier against pathogen invasion (13, 14). Upon viral challenge, host pattern recognition receptors detect viral pathogen-associated molecular patterns, eliciting the phosphorylation and activation of TBK1, which subsequently phosphorylates IRF3 (15, 16). The phosphorylated IRF3 then translocates into the nucleus to instigate the transcriptional induction of interferon-β (IFN-β) and interferon-stimulated genes (ISGs), establishing an antiviral state within host cells (17). To establish productive infection, CoVs have evolved sophisticated strategies to antagonize the host innate immunity at multiple steps (18–20). In contrast, the host encodes a repertoire of antiviral proteins that counteract viral replication by regulating the innate immune response, and identifying such host factors and their underlying antiviral mechanisms is pivotal to unraveling the viral-host interaction network and developing novel antiviral strategies (21, 22).

DEAD-box RNA helicases are a highly conserved family of cellular proteins that play essential roles in RNA metabolism, including transcription, splicing, and translation (23–26). DEAD-box RNA helicase 21 (DDX21), a well-characterized member of the DEAD-box RNA helicase family, serves as a key regulator of multiple fundamental cellular processes, including RNA metabolism, ribosome biogenesis, transcriptional regulation, and genomic stability, and has been shown to restrict the replication of a variety of RNA and DNA viruses (27–30). Our previous study identified an interaction between DDX21 and the N protein of PDCoV using co-immunoprecipitation coupled with mass spectrometry (31), but the role of DDX21 in PDCoV infection remains unclear.

In this study, we found that DDX21 inhibits PDCoV replication in multiple cell types, and this phenotype coincides with elevated interferon responses. Furthermore, we identified that the N-terminal domain (NTD) of PDCoV N binds to the 1–217 amino acid region of DDX21 and drives DDX21 translocation from the nucleus to the cytoplasm. Additionally, DDX21 displayed antiviral activity against multiple CoVs from different genera. This study enriches our understanding of the CoV-host innate immune interactions and provides potential molecular targets for the development of antiviral strategies against CoV infections.

## MATERIALS AND METHODS

### Cells and viruses

PK15, IPEC-J2, HeLa, Vero, LLC-PK1, DF-1, H1299, NCTC, and HEK293T cells were maintained in our laboratory and cultured in Dulbecco’s modified Eagle medium (DMEM) supplemented with 10% fetal bovine serum (FBS, Transgene Biotech) at 37°C with 5% CO_2_ incubation. The DDX21 knockout HeLa cell line was kindly provided by Prof. Yingjie Sun from Shanghai Veterinary Research Institute and propagated in DMEM supplemented with 10% FBS (Transgene Biotech) at 37°C with 5% CO_2_ (32). PDCoV strain CH/JXJGS01/2016 (GenBank accession no. KY293677) was isolated from a piglet in 2016 and propagated in LLC-PK1 cells supplemented with 5 µg/mL trypsin at 37°C with 5% CO_2_. PEDV CH/JX/01 strain (GenBank accession no. KX058031) was isolated from a diarrheic piglet in Jiangxi in 2014 and propagated in Vero cells supplemented with 5 µg/mL trypsin at 37°C with 5% CO_2_. MHV-A59 virus (ATCC VR764) is cultured in NCTC cells and stored in our laboratory. The IBV Beaudette strain was purchased from ATCC and propagated in DF-1 cells.

### Antibody and reagents

Rabbit monoclonal anti-DDX21 was kindly provided by Prof. Yingjie Sun from Shanghai Veterinary Research Institute. Mouse anti-PDCoV N monoclonal antibody was generated in our laboratory. Mouse anti-PEDV N monoclonal antibody was kindly provided by Prof. Shaobo Xiao from Huazhong Agricultural University. Rabbit anti-β-actin monoclonal antibody (Cat. No. AC026), rabbit anti-HA (Cat. No. AE036) polyclonal antibody, rabbit anti-TBK1 polyclonal antibody (Cat. No. A13407), rabbit anti-IRF3 polyclonal antibody (Cat. No. A1211), horseradish peroxidase (HRP)-conjugated goat anti-mouse IgG (Cat. No. AS003), and HRP-conjugated goat anti-rabbit IgG (Cat. No. AS014) were purchased from Abclonal (Wuhan, China). Mouse monoclonal anti-Flag (Cat. No. F1804) was purchased from Sigma-Aldrich. Rabbit anti-phospho-TBK1 (Ser172) monoclonal antibody (Clone D52C2, Cat. No. 5483) and rabbit anti-phospho-IRF3 (Ser396) monoclonal antibody (Clone 4D4G, Cat. No. 4947) were purchased from Cell Signaling Technology. Alexa Fluor 488 (Cat. No. A10235)- or 594 (Cat. No. A-11012)-conjugated secondary antibodies were purchased from Thermo Fisher Scientific.

### Generation of mouse anti-IBV N polyclonal antibody

Mouse anti-IBV N polyclonal antibody was prepared in our laboratory. Briefly, the IBV N gene was cloned into the pET-32a vector to construct the recombinant prokaryotic expression plasmid pET-32a-IBV-N. The verified recombinant plasmid was transformed into *Escherichia coli* BL21 competent cells, and the expression of IBV N protein was induced by 0.5 mM IPTG. The expressed recombinant IBV N protein was purified using an Ni-NTA affinity chromatography column. The purified protein (50 ng) was emulsified with complete Freund’s adjuvant and used for the primary immunization of BALB/c mice. Two booster immunizations were further performed on day 14 and day 28 post the initial immunization, with 50 ng of IBV N protein per mouse mixed with incomplete Freund’s adjuvant. Finally, mouse serum was collected and preserved for subsequent experiments.

### Plasmids

The full-length coding sequence (CDS) of porcine *DDX21* (GenBank accession no. XM_005657387.3) was amplified by PCR from cDNA synthesized by reverse transcription of total RNA extracted from PK-15 cells. The CDSs were cloned into the pCMV-3×FLAG vector to generate the porcine DDX21 expression constructs. Flag-tagged human DDX21, DDX21 (D126A), DDX21 (1–125), and DDX21 (127–784) expression plasmids were kindly provided by Prof. Yingjie Sun from Shanghai Veterinary Research Institute. For functional domain analysis, four series of truncated mutants were generated by overlap extension PCR using porcine pCMV-FLAG-DDX21 as the template. All truncated DDX21 mutants were individually cloned into the pCMV-3×FLAG vector. Four truncated porcine DDX21 constructs were generated and designated M1 (Δ aa 1–217), M2 (Δ aa 218–397), M3 (Δ aa 398–581), and M4 (Δ aa 582–784). These plasmids were specifically designed for expressing DDX21 proteins with deletions of distinct functional domains. To investigate the interactions between viral proteins and host factors, the full-length CDSs of the N genes from PEDV, MHV, IBV, and PDCoV were separately cloned into the pCAGGS vector with an incorporated HA tag at the N-terminals. All constructed plasmids were verified by DNA sequencing to ensure sequence accuracy. The primer sequences used for cloning are listed in Table S1.

### Western blotting analysis

Western blotting was performed as described previously (31). Briefly, cells were lysed in RIPA buffer supplemented with protease inhibitor PMSF (Beyotime) for 15 min. The samples were denatured at 100°C for 10 min and then separated in a 10% sodium dodecyl sulfate polyacrylamide gel electrophoresis (SDS-PAGE) and transferred onto polyvinylidene difluoride membranes (Millipore). After washing in PBS three times, the membrane was blocked with 5% skimmed milk. After blocking, the membrane was sequentially incubated with primary and secondary antibodies. Protein bands were visualized using an enhanced chemiluminescence (ECL) solution (Cat. No. SB-WB012, ShareBio), and grayscale analysis was performed using ImageJ software.

### Immunofluorescence assay

Cells were fixed with 4% formaldehyde for 30 min at room temperature. After fixation, the cells were washed three times with phosphate-buffered saline (PBS), permeabilized with 0.05% Triton X-100 in PBS for 10 min at room temperature, and washed three times again with PBS. Blocking was performed using 5% bovine serum albumin (BSA) in PBS at 37°C for 30 min. The cells were then incubated with primary antibodies for 1 h, followed by washing with PBS and incubation with Alexa Fluor 488- and 594-conjugated secondary antibodies (Thermo Fisher Scientific) for 1 h at 37°C in the dark. Afterward, the cells were stained with DAPI (Cat. No. C1005, Beyotime), mounted with glycerin, and observed under a confocal microscope (Olympus). Image analysis was performed using ImageJ.

### Co-IP assay

For the coimmunoprecipitation (Co-IP) assay, HEK293T cells were transfected with the indicated plasmids for 24 h. Cells were harvested and lysed in NP-40 lysis buffer containing protease inhibitor PMSF (Beyotime). After centrifugation at 12,000 × *g* for 10 min at 4°C, the supernatants were incubated with antibodies prebound to Thermo Scientific Pierce Protein A/G magnetic beads (Cat. No. 88802) at 4°C for 8 h. The beads were washed three times with NP-40 buffer, boiled in protein loading buffer, and subjected to SDS-PAGE followed by Western blotting with the indicated antibodies.

### Quantitative real-time reverse transcription PCR

Total RNA was extracted from cells using TRIzol reagent according to the manufacturer’s instructions (Cat. No. 9108, TaKaRa). First-strand cDNA was synthesized using the First-Strand cDNA Synthesis Kit (Cat. No. AT311, TransGen Biotech). The quantitative PCR (qPCR) was performed in a final volume of 20 μL containing 10 μL Universal SYBR qPCR Master Mix (Cat. No. Q711, Vazyme Biotech), 0.5 μL cDNA template, and 10 μM forward and reverse primers. The thermal cycling conditions were as follows: 5 min at 95°C, followed by 40 cycles of 10 s at 95°C and 30 s at 60°C. All experiments were performed in triplicate using the ABI 7500 Real-Time PCR System (Applied Biosystems). Relative gene expression levels were calculated using the 2^(−ΔΔCt) method and normalized to β-actin. The primer sequences used for quantitative reverse transcription PCR (RT-qPCR) are listed in Table S1.

### Statistical analysis

The data are presented as the mean ± standard deviation (SD). Statistical significance between two groups was assessed using a two-tailed independent Student’s *t*-test using GraphPad Prism 8.0.1. Statistical differences between groups were considered statistically significant when the *P* value was lower than 0.05 (*, *P* < 0.05; **, *P* < 0.01; ***, *P* < 0.001; ****, *P* < 0.0001).

## RESULTS

### DDX21 exerts suppressive effects on PDCoV replication

**Fig 1:**
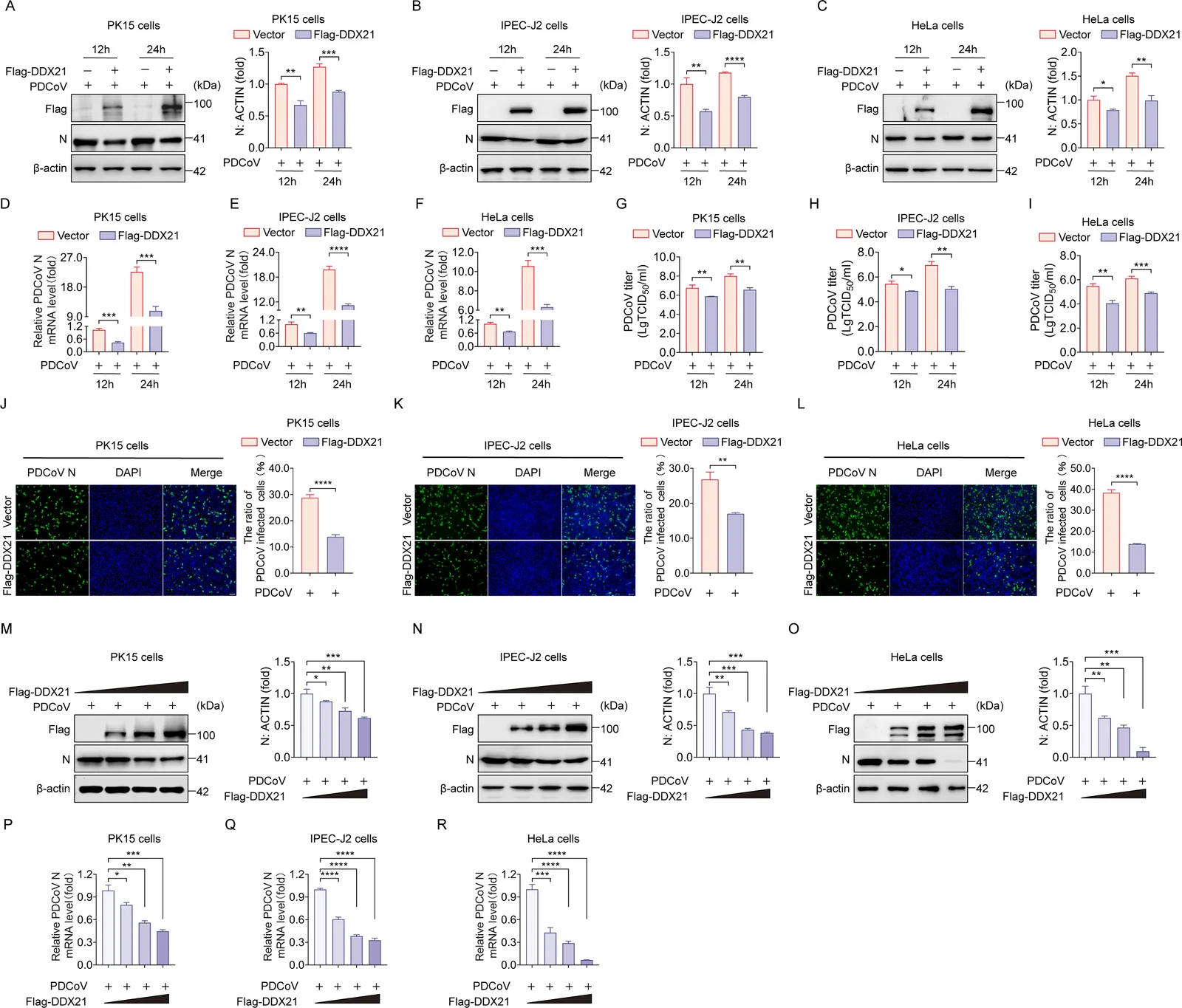
Overexpression of DDX21 inhibits PDCoV replication *in vitro*. (**A–C**) Immunoblotting analysis of PDCoV N protein abundance in PK15 (**A**), IPEC-J2 (**B**), and HeLa (**C**) cells. Cells were transfected or mock-transfected with DDX21 for 24 h and then infected with 0.1 MOI of PDCoV for 12 or 24 h. Anti-Flag antibody detected exogenous DDX21; β-actin served as a loading control. Bar graphs show relative quantification of PDCoV N protein normalized to β-actin. (**D–F**) Quantification of intracellular PDCoV N transcripts in PK15 (**D**), IPEC-J2 (**E**), and HeLa (**F**) cells treated as in panels A–C. (**G–I**) Determination of infectious PDCoV titers in cell culture supernatants via TCID₅₀ assay from PK15 (**G**), IPEC-J2 (**H**), and HeLa (**I**) cells treated as in panels A–C. (**J–L**) Immunofluorescence assay analysis of PDCoV-infected PK15 (**J**), IPEC-J2 (**K**), and HeLa (**L**) cells transfected with empty vector or Flag-DDX21. PDCoV N protein was stained green; cell nuclei were counterstained with DAPI (blue). Scale bar, 100 μm. Bar plots quantify the percentage of PDCoV N-positive infected cells in each group. (**M–O**) DDX21 restrains PDCoV propagation in a dose-dependent manner in PK15 (**M**), IPEC-J2 (**N**), and HeLa (**O**) cells. Cells were transfected with serially elevated Flag-DDX21 plasmids before PDCoV infection. Immunoblotting was performed to detect Flag-DDX21, N protein, and β-actin. Bar graphs show quantification of relative PDCoV N protein levels normalized to β-actin. (**P–R**) RT-qPCR quantification of relative PDCoV N transcript levels in PK15 (**P**), IPEC-J2 (**Q**), and HeLa (**R**) cells transfected with increasing doses of Flag-DDX21. Data are presented as mean ± SD of three independent replicates. *, *P* < 0.05; **, *P* < 0.01; ***, *P* < 0.001; ****, *P* < 0.0001.

To screen for host factors involved in PDCoV replication, we previously conducted Co-IP coupled with mass spectrometry and identified that DDX21 can interact with PDCoV N protein (31). To evaluate the regulatory role of DDX21 during PDCoV infection, PK15, IPEC-J2, and HeLa cells were transfected with 1 μg of Flag-DDX21 plasmid. At 24 h post-transfection, cells were infected with PDCoV at a multiplicity of infection (MOI) of 0.1 for 12 and 24 h, respectively. Cells were then harvested for detection of PDCoV N protein expression, and supernatants were collected for viral titer determination. The results demonstrated that PDCoV N protein expression was significantly reduced in DDX21-overexpressing PK15, IPEC-J2, and HeLa cells (Fig. 1A through C). In addition, PDCoV N mRNA transcripts were also significantly decreased in PK15, IPEC-J2, and HeLa cells transfected with DDX21 (Fig. 1D through F). Consistent with these observations, the infectious viral titer of PDCoV was significantly lower in DDX21-overexpressing cells than in empty vector-transfected control cells at 12 and 24 h post-infection (hpi) (Fig. 1G through I). Compared with the mock-transfected cells, there was obviously weaker fluorescence intensity in DDX21-overexpressing cells in PK15, IPEC-J2, and HeLa cells at 12 hpi (Fig. 1J through L). To determine whether DDX21 specifically inhibits PDCoV replication, we performed a dose-dependent assay. The results revealed that both the protein and mRNA levels of PDCoV N were gradually reduced in a DDX21 dose-dependent manner in PK15, IPEC-J2, and HeLa cells (Fig. 1M through R). Collectively, these data demonstrate that DDX21 exerts a conserved, cell type-independent suppressive effect on PDCoV replication.

### Knockout of DDX21 promotes PDCoV replication

**Fig 2:**
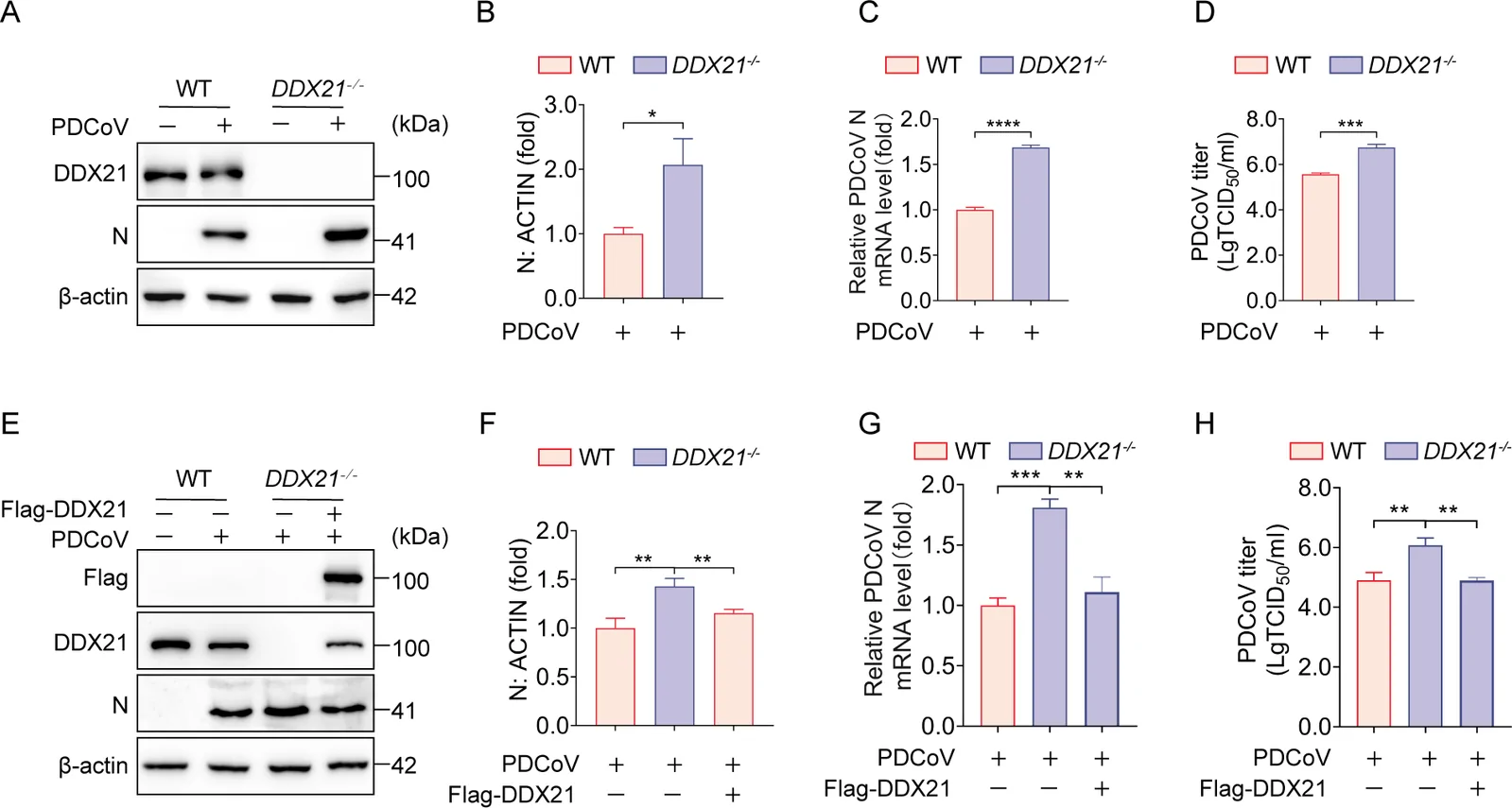
Knockout of DDX21 enhances PDCoV replication**.** (**A**) Immunoblotting analysis of PDCoV N protein abundance in PDCoV-infected WT and *DDX21^−/^*^−^ HeLa cells; β-actin serves as a loading control. (**B**) Densitometric analysis of normalized PDCoV N protein abundance from panel A. (**C**) RT-qPCR quantification of intracellular PDCoV N mRNA in infected WT and *DDX21^−/^*^−^ HeLa cells. (**D**) Infectious PDCoV titers from cell culture supernatants of infected WT and *DDX21^−/^*^−^ HeLa cells. (**E**) DDX21 was transfected into WT and *DDX21^−/^*^−^ HeLa cells and then infected with PDCoV (MOI = 0.1) for 12 h, followed by Western blotting analysis. (**F–H**) Corresponding quantification of PDCoV N protein (**F**), PDCoV N mRNA (**G**), and extracellular infectious virus titers (**H**) for the rescue experiments in panel E. Data are presented as mean ± SD from three independent biological experiments. *, *P* < 0.05; **, *P* < 0.01; ***, *P* < 0.001; ****, *P* < 0.0001.

Having verified that DDX21 overexpression suppresses PDCoV replication *in vitro*, we next employed *DDX21^−/^*^−^ HeLa cells to further characterize the regulatory role of DDX21 during PDCoV infection. As presented in Fig. 2A through C, PDCoV N protein and viral mRNA levels were significantly elevated in *DDX21^−/^*^−^ HeLa cells compared with wild-type (WT) cells at 12 hpi. In accordance with these findings, the infectious viral titer of PDCoV was also markedly increased in *DDX21^−/^*^−^ HeLa cells (Fig. 2D). Furthermore, rescue experiments via ectopic expression of DDX21 in *DDX21^−/^*^−^ HeLa cells followed by PDCoV infection (MOI = 0.1) for 12 h demonstrated that restoration of DDX21 expression significantly reversed the promotion of PDCoV replication induced by DDX21 deficiency (Fig. 2E through H). These results prove that knockout of DDX21 promotes PDCoV replication *in vitro*.

### PDCoV infection reduces DDX21 expression

**Fig 3:**
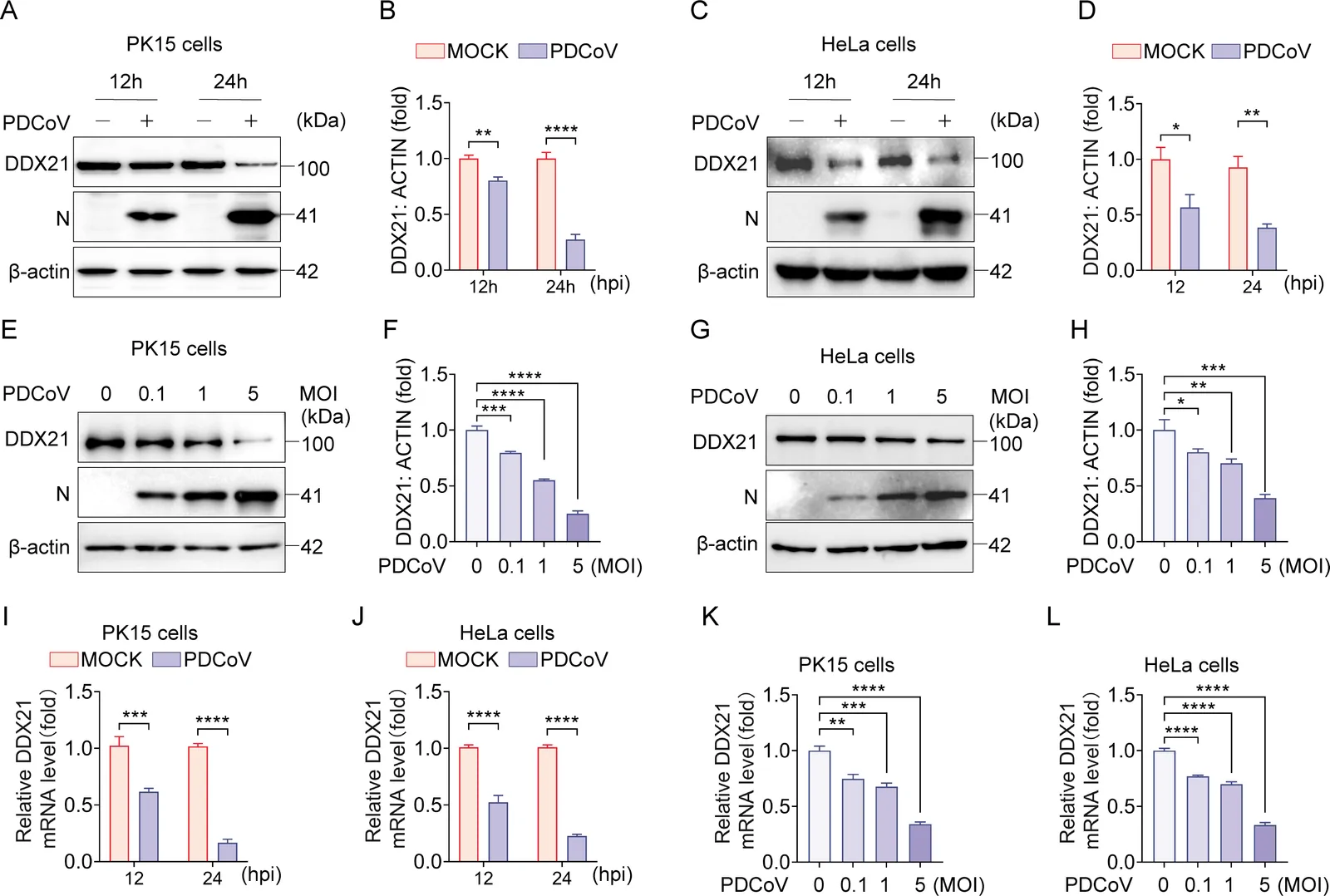
PDCoV infection downregulates the expression of DDX21**.** (**A–D**) Immunoblot and densitometry for DDX21 and PDCoV N protein in PK15 (**A, B**) and HeLa (**C, D**) cells at 12 and 24 hpi; β-actin served as a loading control. DDX21 protein abundance was normalized to β-actin and compared with mock controls. (**E–H**) Dose-gradient infection with PDCoV at MOIs of 0, 0.1, 1, and 5. Immunoblots and corresponding quantification of DDX21 protein in PK15 (**E, F**) and HeLa (**G, H**) cells. (**I, J**) DDX21 mRNA abundance in PK15 (**I**) and HeLa (**J**) cells infected with PDCoV at 12 and 24 hpi. (**K, L**) DDX21 mRNA abundance in PK15 (**K**) and HeLa (**L**) cells infected with PDCoV at MOIs of 0, 0.1, 1, and 5 was measured by RT-qPCR. Data are presented as mean ± SD from three independent biological experiments. *, *P* < 0.05; **, *P* < 0.01; ***, *P* < 0.001; ****, *P* < 0.0001.

The above results have demonstrated that DDX21 has an antiviral effect on PDCoV replication. To determine the expression level of DDX21 during PDCoV infection, we analyzed the endogenous expression of DDX21 in PK15 and HeLa cells upon PDCoV infection. The protein level of DDX21 was significantly decreased in PDCoV-infected PK15 and HeLa cells at 12 and 24 hpi, which was further validated by the relative density ratio analysis of DDX21 normalized to β-actin (the corresponding internal reference) (Fig. 3A through D). To further verify the expression dynamics of DDX21 during PDCoV infection, we conducted a dose-dependent assay. As presented in Fig. 3E through H, the decrease in DDX21 protein became more pronounced with increasing PDCoV infection doses in PK15 and HeLa cells. Similarly, the mRNA level of DDX21 was also markedly decreased at 12 and 24 hpi in PK15 and HeLa cells (Fig. 3I through J). Furthermore, downregulation of the DDX21 mRNA occurred in a dose-dependent manner in PDCoV-infected PK15 and HeLa cells at MOIs of 0.1, 1, and 5, respectively (Fig. 3K and L). These results indicate that PDCoV infection can significantly reduce DDX21 expression at both the protein and mRNA levels.

### The aa 1–217 of DDX21 interacts with the NTD of PDCoV N

**Fig 4:**
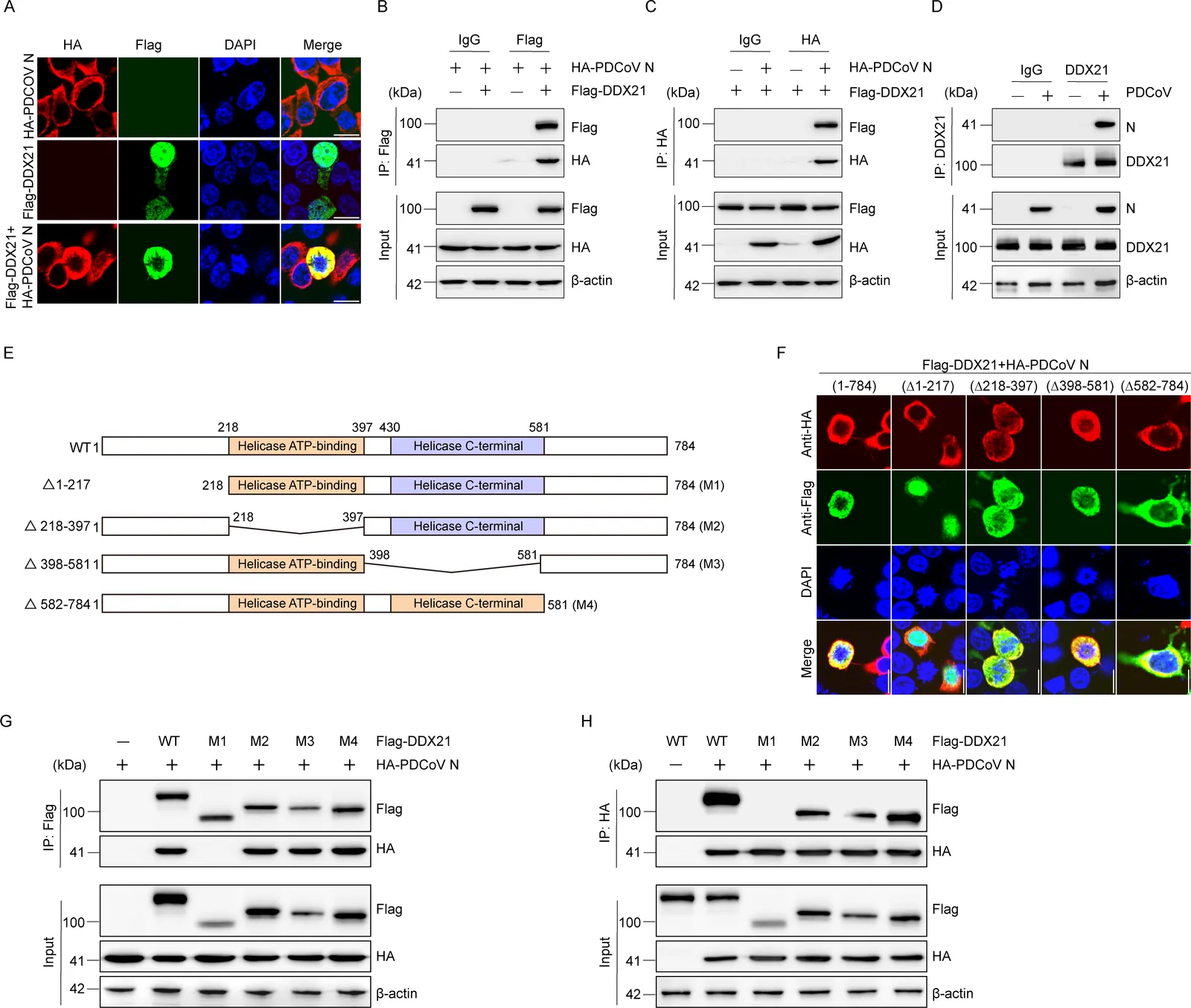
The aa 1–217 of DDX21 interacts with PDCoV N**.** (**A**) Analysis of the subcellular colocalization of DDX21 (green) and PDCoV N (red) in transfected cells by confocal immunofluorescence microscopy. Nuclei were stained with DAPI (blue). Scale bar, 10 μm. (**B, C**) Bidirectional Co-IP assays confirm DDX21 interacts with PDCoV N. Anti-Flag (**B**) and anti-HA (**C**) antibodies were used for reciprocal pulldowns, with nonspecific IgG as a negative control. (**D**) Cell lysates were immunoprecipitated with anti-DDX21 antibody or control IgG, followed by immunoblotting to detect bound endogenous PDCoV N protein. (**E**) Schematic diagrams of full-length and truncated mutants of the DDX21 protein. (**F**) Laser confocal microscopy analysis of the colocalization of the viral N protein with full-length DDX21 or each DDX21 truncated mutant. Nuclei were stained with DAPI. Scale bar, 10 μm. (**G, H**) The expression plasmids of full-length or truncated mutants of DDX21 and HA-PDCoV N were co-transfected into HEK293T cells for 24 h. Cell lysates were immunoprecipitated with anti-Flag (**G**) or anti-HA (**H**) antibody and analyzed with immunoblotting.

Previously, we conducted Co-IP coupled with mass spectrometry and identified that DDX21 may interact with the PDCoV N protein (31). To confirm the interaction relationship between DDX21 and PDCoV N, confocal microscopy and Co-IP assay were performed. As shown in Fig. 4A, DDX21 could spatially overlap with PDCoV N in cells co-transfected with Flag-DDX21 and HA-PDCoV-N, demonstrating that the two proteins were colocalized and capable of interacting with each other. To further verify the interaction between DDX21 and PDCoV N, HEK293T cells co-expressing FLAG-DDX21 and HA-PDCoV-N were harvested for immunoprecipitation with anti-FLAG, anti-HA, or mouse IgG antibodies, and precipitated products were detected by Western blotting using corresponding antibodies. The Co-IP results indicate that the DDX21 protein could interact with the PDCoV N protein (Fig. 4B and C). Furthermore, endogenous Co-IP assays were also carried out to verify the interaction between DDX21 and the PDCoV N protein. PK15 cells were infected with PDCoV at an MOI of 0.1 for 12 h, after which cell lysates were immunoprecipitated using anti-DDX21 antibody or control IgG. The precipitated products were then subjected to Western blotting analysis with corresponding antibodies. As presented in Fig. 4D, endogenous DDX21 interacts with PDCoV N protein under PDCoV infection conditions.

DDX21 protein consists of four functional domains: the N-terminal domain (residues 1–217), helicase core domain (residues 218–397), RHII/Gu C-terminal domain (residues 398–581), and C-terminal tail (residues 582–784) (Fig. 4E). To determine which domain of DDX21 is required for its interaction with the PDCoV N protein, we constructed four truncated DDX21 mutants, designated M1 (DDX21 lacking residues 1–217), M2 (DDX21 lacking residues 218–397), M3 (DDX21 lacking residues 398–581), and M4 (DDX21 lacking residues 582–784). The interactions between each mutant and the PDCoV N protein were examined by confocal microscopy and Co-IP. Both assays showed that only WT DDX21 and mutants retaining the 1–217 aa domain displayed a specific interaction with the PDCoV N protein, whereas all mutants lacking the 1–217 aa region failed to bind to the PDCoV N protein (Fig. 4F through H). Moreover, the interaction between the N protein and DDX21 drives DDX21 translocation from the nucleus to the cytoplasm. These results indicate that the 1–217 aa region of DDX21 represents the critical domain responsible for interaction with the PDCoV N protein.

**Fig 5:**
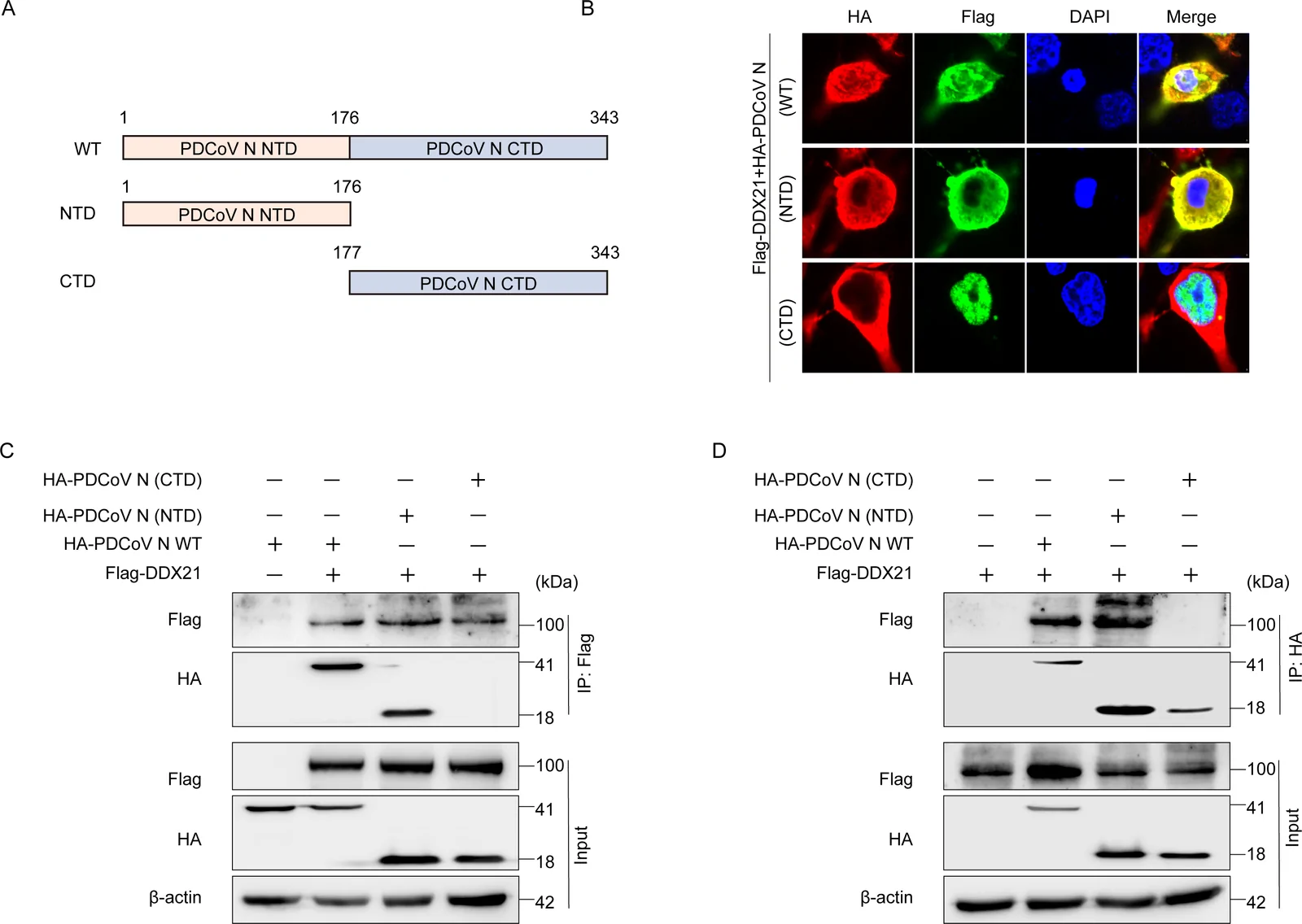
The NTD of PDCoV N interacts with DDX21**.** (**A**) Schematic diagrams of full-length and truncation mutants of PDCoV N protein. (**B**) Confocal immunofluorescence showing subcellular colocalization between ectopically expressed DDX21 (green) and each PDCoV N truncation mutant (red). Nuclei were stained with DAPI (blue). (**C, D**) The expression plasmids of full-length or truncated mutants of PDCoV N and Flag-DDX21 were co-transfected into HEK293T cells for 24 h. Cell lysates were immunoprecipitated with anti-Flag (**C**) or anti-HA (**D**) antibody and analyzed with immunoblotting.

To further identify the domain of PDCoV N protein responsible for the interaction with DDX21, we constructed two truncated mutants corresponding to the N-terminal domain (NTD) and C-terminal domain (CTD) of PDCoV N (Fig. 5A). Confocal microscopy assay showed that both full-length PDCoV N and the NTD mutant exhibited obvious colocalization with DDX21, whereas the CTD mutant did not (Fig. 5B). Consistently, Co-IP results confirmed that full-length PDCoV N and the NTD mutant interacted with DDX21, while the CTD mutant failed to interact with DDX21 (Fig. 5C and D). Collectively, these results demonstrate that the NTD of the PDCoV N protein is the critical domain mediating its interaction with DDX21.

### DDX21 is associated with elevated IFN responses during PDCoV infection

**Fig 6:**
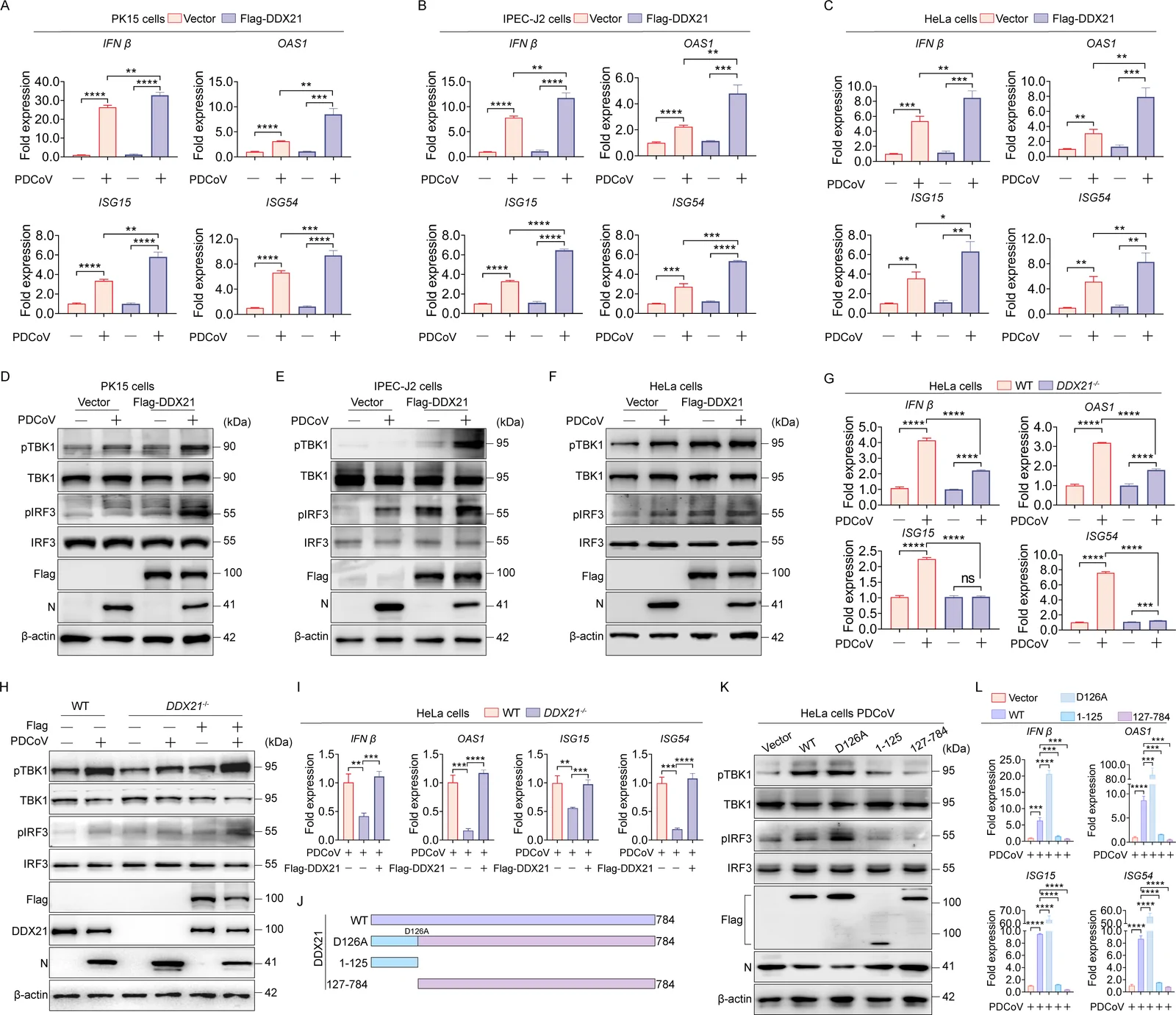
DDX21 is associated with elevated IFN responses during PDCoV infection**.** (**A–C**) Empty vector and Flag-DDX21-transfected PK15 (**A**), IPEC-J2 (**B**), and HeLa (**C**) cells were infected or mock-infected with PDCoV for 12 hpi. Cells were harvested for RT-qPCR analysis to determine the mRNA levels of IFN-β, OAS1, ISG15, and ISG54. (**D–F**) PK15 (**D**), IPEC-J2 (**E**), and HeLa (**F**) cells were transfected with DDX21 or the empty vector and then infected or mock-infected with PDCoV (MOI = 0.1) for 12 h. Cell lysates were subjected to immunoblotting to detect the protein levels of pTBK1, TBK1, pIRF3, IRF3, DDX21, PDCoV N, and β-actin. (**G**) WT and *DDX21^−/^*^−^ HeLa cells were infected or mock-infected with PDCoV for 12 h, respectively. Cell samples were collected for RT-qPCR to assess the mRNA levels of IFN-β, OAS1, ISG15, and ISG54. (**H**) WT, *DDX21^−/^*^−^, and DDX21-rescued *DDX21^−/^*^−^ HeLa cells were infected or mock-infected with PDCoV for 12 h. Cell lysates were subjected to immunoblotting to detect the protein levels of pTBK1, TBK1, pIRF3, IRF3, Flag-DDX21, DDX21, PDCoV N, and β-actin. (**I**) WT, *DDX21^−/^*^−^, and DDX21-rescued *DDX21^−/^*^−^ HeLa cells were infected with PDCoV for 12 h. Cells were collected for RT-qPCR to evaluate the mRNA expression of IFN-β, OAS1, ISG15, and ISG54. (**J**) Schematic illustration of DDX21 WT and its caspase-cleaved forms. (**K**) Western blotting analysis of TBK1/IRF3 phosphorylation, DDX21, PDCoV N, and β-actin in PDCoV-infected HeLa cells transfected with empty vector, WT DDX21, DDX21 (D126A), DDX21 (1–125), or DDX21 (127–784). (**L**) RT-qPCR measurement of IFNβ, OAS1, ISG15, and ISG54 transcript levels corresponding to panel K. Data are presented as mean ± SD from three independent biological experiments. ns, no significance; **, *P* < 0.01; ***, *P* < 0.001; ****, *P* < 0.0001.

DEAD (Asp-Glu-Ala-Asp) box RNA helicases have been proven to contribute to antiviral innate immunity (33). To elucidate the molecular mechanism by which DDX21 inhibits PDCoV replication, we investigated the correlation between DDX21 and type I IFN signaling during PDCoV infection. We first examined the role of exogenous DDX21 in the IFN response upon PDCoV infection. The results showed that the mRNA expression levels of IFN-β and its downstream ISGs, including OAS1, ISG15, and ISG54, were significantly higher at 12 hpi in DDX21-overexpression cells compared with WT cells (Fig. 6A through C). Consistent with the enhanced ISG expression, Western blotting analysis revealed that DDX21 overexpression markedly promoted the phosphorylation of TBK1 and IRF3 following PDCoV infection in PK15, IPEC-J2, and HeLa cells (Fig. 6D through F). Concomitantly, the protein level of PDCoV N protein was drastically reduced in DDX21-overexpressing cells, confirming the robust antiviral activity of DDX21 (Fig. 6D through F). We also found that in *DDX21^−/^*^−^ HeLa cells, the mRNA expression levels of IFN-β and its downstream IFN-stimulated genes were significantly lower at 12 hpi compared with WT cells (Fig. 6G). These findings indicate that DDX21 is a host restriction factor that correlates with efficient induction of the host type I IFN response upon PDCoV infection.

We next performed a rescue experiment to further characterize the phenotypes linked to DDX21-mediated inhibition of PDCoV replication. The results showed that overexpression of DDX21 in *DDX21^−/−^* HeLa cells markedly suppressed PDCoV replication (Fig. 6H). Western blotting analysis also revealed that restoring DDX21 expression markedly restored the phosphorylation levels of TBK1 and IRF3 during PDCoV infection (Fig. 6H)**.** Consistently, mRNA levels of IFN-β and the ISGs were fully restored to those in infected WT cells (Fig. 6I). Collectively, these results demonstrated that DDX21 is positively correlated with enhanced IFN responses accompanied by TBK1-IRF3 pathway activation during PDCoV infection.

DDX21 undergoes caspase-dependent cleavage at D126 to promote its translocation from the nucleus to the cytoplasm in response to late virus infection (32). To dissect the distinct immunomodulatory functions of DDX21, we engineered a panel of DDX21 constructs encompassing the N-terminal fragment (1–125), the cleaved C-terminal fragment (127–784), WT DDX21, and cleavage-resistant DDX21 (D126A) (Fig. 6J). Immunoblotting analysis of PDCoV-infected HeLa cells revealed that ectopic expression of either WT DDX21 or DDX21 (D126A) robustly augmented phosphorylation of TBK1 and IRF3 (Fig. 6K). This amplified IFN response signature coincided with suppressed PDCoV replication (Fig. 6K). Conversely, the cleaved DDX21 could not trigger the IFN responses in PDCoV-infected cells (Fig. 6K). Consistent with the immunoblotting phenotypes, RT-qPCR quantification of IFN-β and ISGs further verified that WT and DDX21 (D126A) correlated with amplified PDCoV-induced antiviral transcriptional responses, while the cleaved DDX21 variants failed to boost IFN and ISG expression (Fig. 6L).

### DDX21 exerts antiviral activity against several CoVs

**Fig 7:**
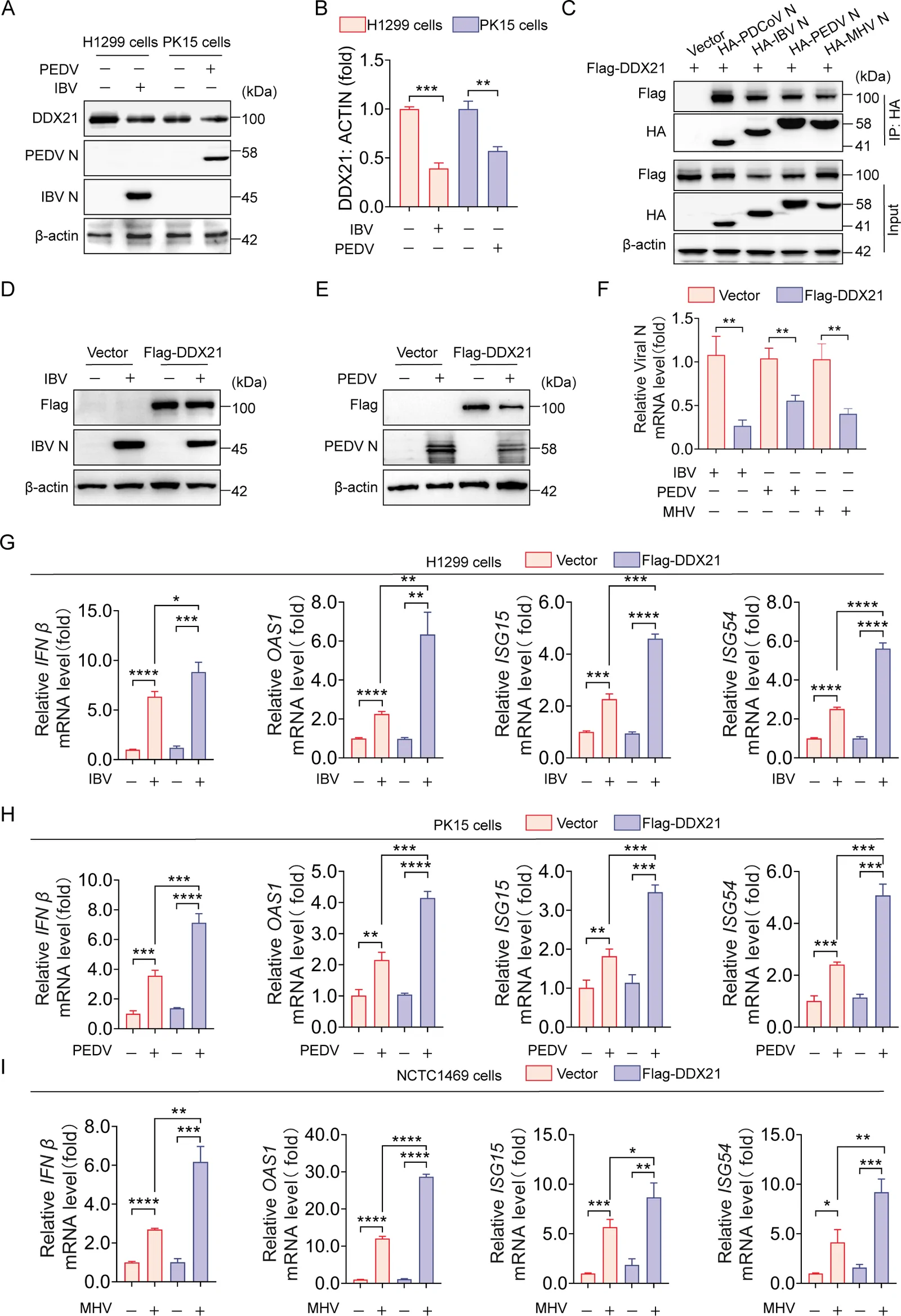
DDX21 exhibits antiviral activity against multiple CoVs**.** (**A**) Western blotting showing endogenous DDX21 protein levels in IBV-infected H1299 cells and PEDV-infected PK15 cells; β-actin served as a loading control. (**B**) Relative band intensity ratios of DDX21 to β-actin in panel **A** were quantified and normalized to the control group. (**C**) Co-IP assay demonstrating an interaction between DDX21 and viral N proteins of four CoVs in transfected HEK293T cells. (**D, E**) Western blotting analysis of viral N protein abundance in IBV-infected H1299 (**D**) and PEDV-infected PK15 (**E**) cells transfected with the empty vector or Flag-DDX21 for 24 h. (**F**) RT-qPCR analysis of viral N transcripts in DDX21-overexpressing H1299 (IBV), PK15 (PEDV), and NCTC1469 (MHV) cells at 12 hpi. (**G–I**) RT-qPCR quantification of IFN-β, OAS1, ISG15, and ISG54 transcripts in mock- or CoV-infected H1299 (IBV, **G**), PK15 (PEDV, **H**), and NCTC1469 (MHV, **I**) cells with vector or Flag-DDX21 overexpression. Data are presented as mean ± SD from three independent biological experiments. *, *P* < 0.05; **, *P* < 0.01; ***, *P* < 0.001; ****, *P* < 0.0001.

The above results have confirmed that DDX21 can inhibit PDCoV replication. We then tested whether DDX21 exhibits antiviral activity against other CoVs. We observed that endogenous DDX21 protein was significantly downregulated during *gammacoronavirus* IBV and *alphacoronavirus* PEDV infection (Fig. 7A and B), implying that CoVs have evolved a conserved strategy to antagonize the antiviral function of DDX21. Then, we also evaluated the interaction between DDX21 and the N proteins of multiple CoVs spanning distinct genera. Co-IP assays demonstrated that DDX21 associates with the N proteins of PEDV, MHV, and IBV (Fig. 7C), indicating that this interaction is conserved among the tested CoVs. Moreover, we assessed the effects of DDX21 on the replication of different CoVs. Overexpression of DDX21 in H1299 and PK15 cells significantly suppressed IBV and PEDV replication at the protein and mRNA levels, respectively (Fig. 7D through F). Due to the unavailability of an MHV-specific antibody, we evaluated the inhibitory effect of DDX21 on MHV replication at the mRNA level. Ectopic expression of DDX21 in NCTC cells markedly reduced MHV mRNA abundance (Fig. 7F). Concomitantly, DDX21-overexpressing cells exhibited a significantly elevated expression of IFN-β and ISGs following infection with IBV, PEDV, or MHV, compared with the control cells (Fig. 7G through I). Collectively, these findings establish that DDX21 functions as an antiviral factor against CoVs.

## DISCUSSION

**Fig 8:**
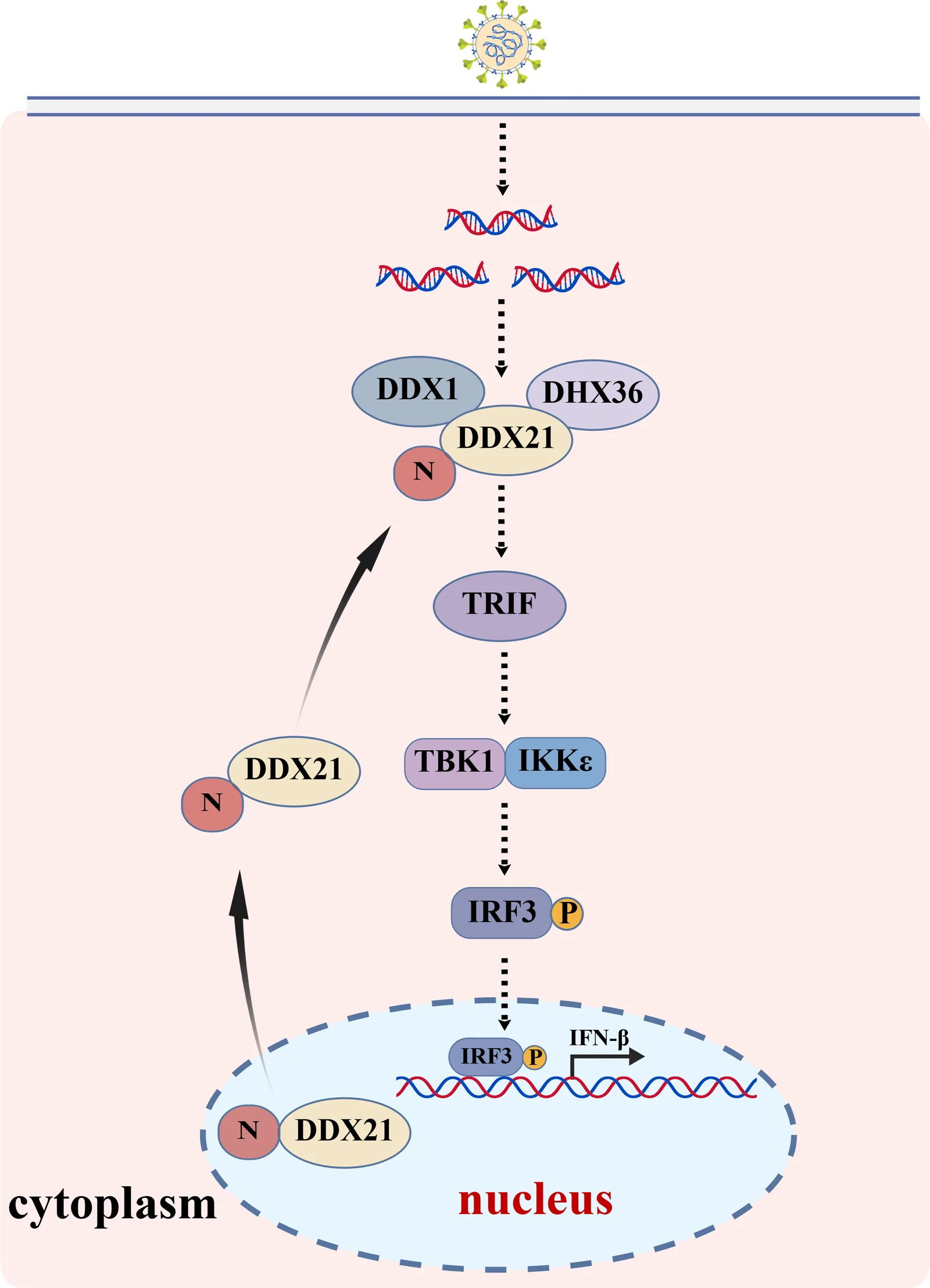
A proposed model of CoV N-DDX21 interaction regulating the IFN pathway**.** During CoV infection, viral nucleic acids enter the cytoplasm. Viral N protein interacts with nuclear DDX21 to facilitate its nuclear export. The cytoplasmic N-DDX21 complex further assembles with DDX1 and DHX36. This complex recruits TRIF to activate the downstream signaling pathway. Activated TBK1 and IKKε induce IRF3 phosphorylation, and phosphorylated IRF3 translocates into the nucleus to initiate IFN-β transcription.

CoVs are a large group of enveloped RNA viruses that pose a persistent and severe threat to both human and animal health worldwide. The continuous emergence of viral variants highlights the urgent need to identify conserved host antiviral factors that can serve as potential therapeutic targets against CoV infection. DDX21 assembles into a heteromeric complex with DDX1 and DHX36, which recognizes dsRNA and subsequently associates with the adapter TRIF to activate downstream signals, mounting an autonomous antiviral immune response (34). Our previous study identified a possible interaction between DDX21 and the N protein of PDCoV (31). However, the molecular mechanism underlying this interaction and its functional role in PDCoV replication remain to be elucidated. In the present study, we systematically demonstrate that DDX21 acts as a critical host antiviral factor that restricts PDCoV infection through interaction with the PDCoV N protein and induction of type I IFN response (Fig. 8).

Previous studies have identified that DDX21 shows antiviral activities against multiple viruses. For example, DDX21 restricts influenza A virus replication by binding to PB1 and inhibiting polymerase assembly (35). DDX21 is also able to suppress foot-and-mouth disease virus propagation by binding to the viral internal ribosome entry site (IRES) (36). DDX21 translocates from the nucleus to the cytoplasm to stimulate the innate immune response against dengue virus infection (37). However, several reports have demonstrated that DDX21 may also dampen host antiviral immunity to facilitate viral propagation. For instance, DDX21 stabilizes the expression of nsp1α, nsp1β, and nucleocapsid proteins of porcine reproductive and respiratory syndrome virus, thereby suppressing IFN-β production and promoting viral replication (38, 39). Similarly, DDX21 enhances PCV3 replication by interacting with the Cap protein and inhibiting IFN responses (40). The above examples show that DDX21 exerts a double-edged-sword role in the regulation of innate immunity upon virus infection. Full-length DDX21 positively regulates IFN-β responses via forming the cytoplasmic DDX1-DDX21-DHX36-TRIF complex, whereas caspase-cleaved truncated DDX21 disrupts this complex and suppresses antiviral immunity (41). Under our experimental PDCoV infection conditions, DDX21 cleavage is minimal, and intact full-length DDX21 represents the major functional role. Our data demonstrate that PDCoV N protein interacts with DDX21 and facilitates its nucleocytoplasmic translocation to augment IFN-β signaling. It is plausible that prolonged or high-dose PDCoV infection may trigger enhanced caspase activation and robust DDX21 cleavage, which in turn restrains innate immune responses.

Viral evasion of host innate immunity is a hallmark of CoV infection, and most CoVs employ multiple strategies to antagonize the IFN signaling pathway, such as inhibition of TBK1/IRF3 activation or degradation of ISG products (42). In this study, PDCoV N interacts with DDX21 and triggers the nuclear-to-cytoplasmic translocation of DDX21, which subsequently initiates IFN responses. As infection progresses, the virus actively reduces the abundance of DDX21 to counteract this host protective antiviral response, thereby favoring viral propagation. The infection-induced downregulation of DDX21 represents an immune antagonism strategy deployed by PDCoV. Moreover, PDCoV N serves as a multifunctional regulator of type I IFN signaling via distinct structural domains. The NTD of PDCoV N interacts with DDX21 to facilitate its nucleocytoplasmic translocation to activate IFN responses at early infection stages. In contrast, the CTD of PDCoV N negatively regulates downstream type I IFN signaling by promoting KPNA2 degradation, which blocks STAT1 nuclear translocation and impairs ISGF3 complex assembly (43). The distinct host targets engaged by separate domains of PDCoV N reflect a layered viral regulatory strategy, under which the NTD initiates upstream IFN activation via DDX21 translocation, whereas the CTD mediates sustained immune suppression by disrupting STAT1 nuclear trafficking to maintain host immune homeostasis.

The NTD of CoV N proteins is a highly conserved domain primarily responsible for RNA binding and protein-protein interactions, which is critical for viral genome packaging and replication (44–46). The N-terminal domain of DDX21 has been shown to mediate protein-protein interactions with other host and viral factors in previous studies (47, 48). Here, we show that DDX21 exerts antiviral effects against several CoVs from different genera. We propose that this antiviral activity is associated with the interaction between DDX21 and CoV N protein, a highly conserved structural protein across CoV genera. In addition, DDX21 is relatively conserved across many host species. Sequence alignment shows that the overall amino acid identity of DDX21 ranges from 66.1% to 99.9% among the examined species. Specifically, porcine DDX21 shares 89.8% sequence identity with human DDX21 and 93.2% identity with bovine DDX21, whereas mouse and chicken DDX21 exhibit relatively low homology (66.1%) (Table S2). The relatively high sequence conservation of DDX21 among multiple species may support the possibility that DDX21 could restrict infection by diverse CoVs, although further work is required to validate this conclusion.

In summary, this study revealed that DDX21 interacts with the NTD of the PDCoV N protein via its N-terminal 1–217 aa domain. This interaction is accompanied by activation of the TBK1-IRF3-mediated type I IFN signaling and elevated expression of IFN-β and ISG, which correlates with the antiviral phenotype of DDX21. We also found that DDX21 exhibits antiviral effects against *Alpha*, *Beta*, *Gamma*, and *Delta* CoVs. These findings provide new insights into the host innate immune responses upon CoV infection and imply that DDX21 might be a promising target for developing anti-CoV strategies.

## Data Availability

Raw Western blot images and complete densitometric quantification data are included in the supplemental material. All other data are contained within the article.

## SUPPLEMENTAL MATERIAL

File S1 jvi.01169-26-s0001.pdf Raw Western blot and complete densitometric quantification data.

Supplemental tables jvi.01169-26-s0002.docx Tables S1 and S2.

ASM does not own the copyrights to Supplemental Material that may be linked to, or accessed through, an article. The authors have granted ASM a non-exclusive, world-wide license to publish the Supplemental Material files. Please contact the corresponding author directly for reuse.
