# Supplementary material for: DEAD-box RNA helicase 21 interacts with the nucleocapsid protein and inhibits porcine deltacoronavirus infection through upregulation of interferon responses
Source: J Virol. 2026 Sep 30;100(10):e01169-26. doi: 10.1128/jvi.01169-26 (PMC13712866; doi:10.1128/jvi.01169-26)
Supplement: Supplementary file 1 [file jvi.01169-26-s0001.pdf]

Fig. 1A β-actin

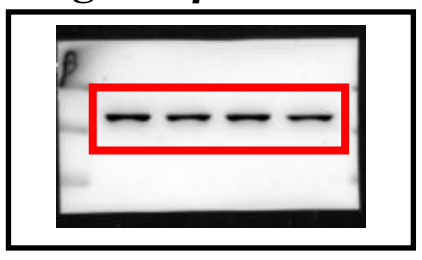

Fig. 1A PDCoV N

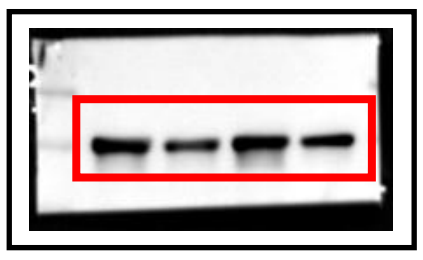

Fig. 1A Flag

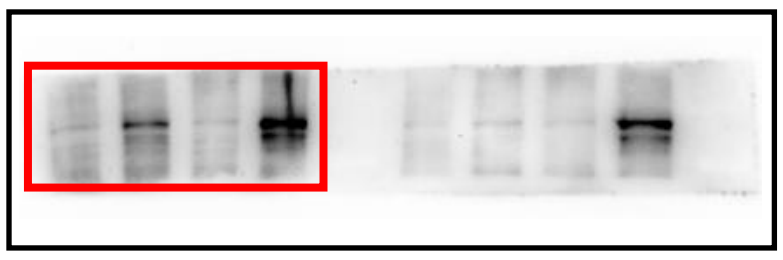

Fig. 1B β-actin

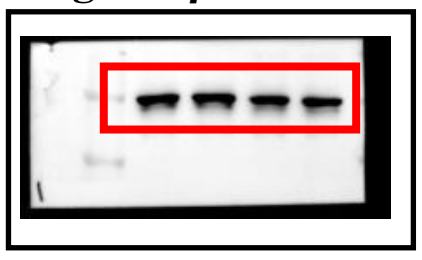

Fig. 1B PDCoV N

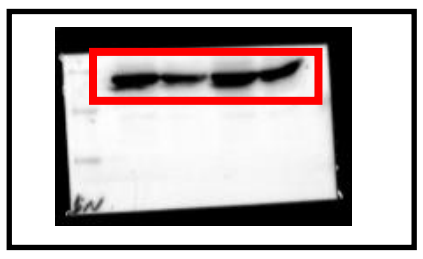

Fig. 1B Flag

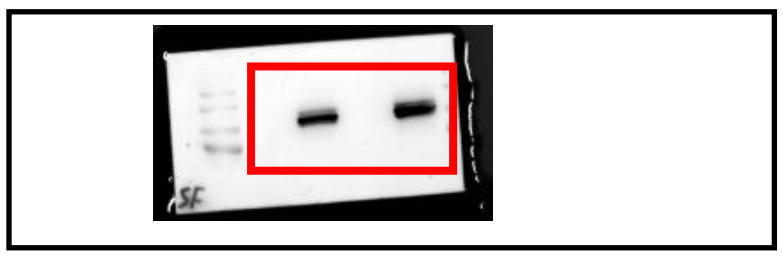

Fig. 1C β-actin

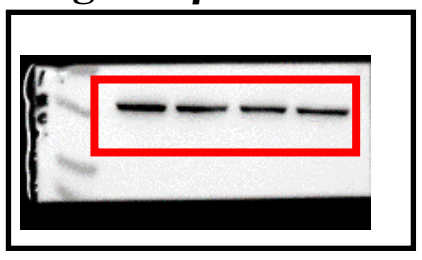

Fig. 1C PDCoV N

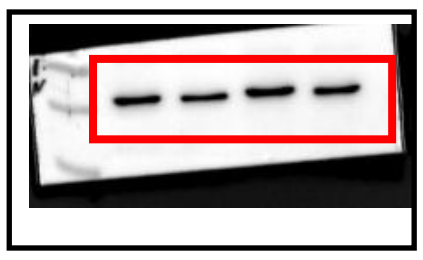

Fig. 1C Flag

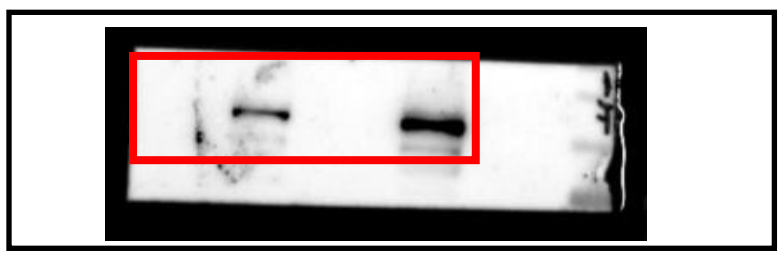

Fig. 1M β-actin

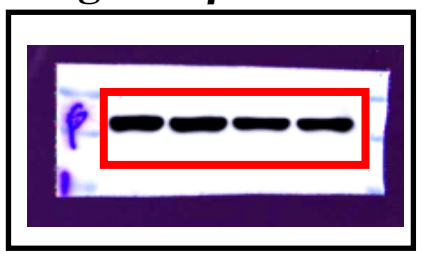

Fig. 1M PDCoV N

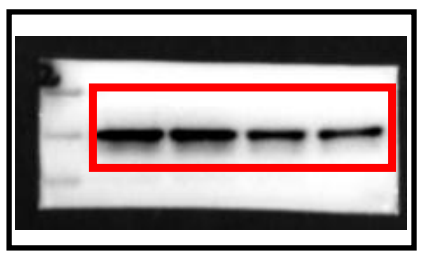

Fig. 1M Flag

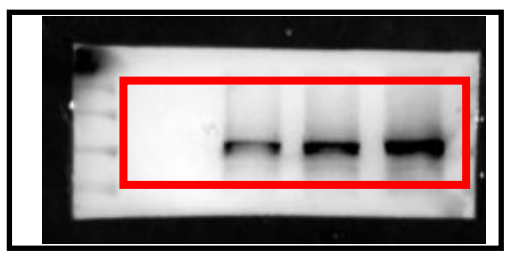

Fig. 1N β-actin

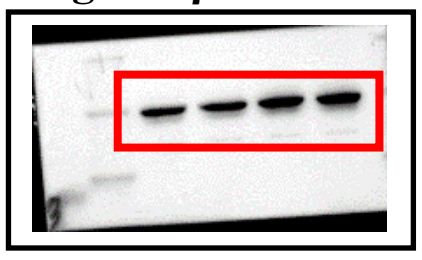

Fig. 1N PDCoV N

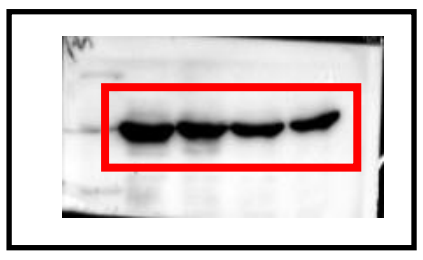

Fig. 1N Flag

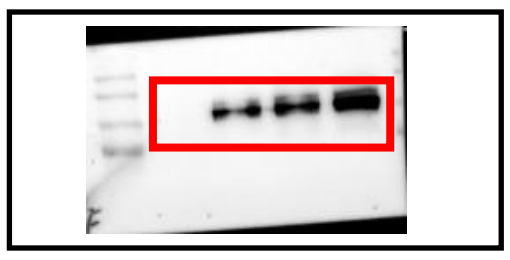

Fig. 1O β-actin

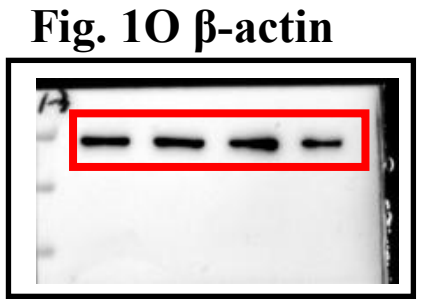

Fig. 1O PDCoV N

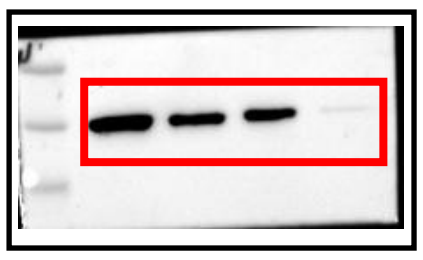

Fig. 1O Flag

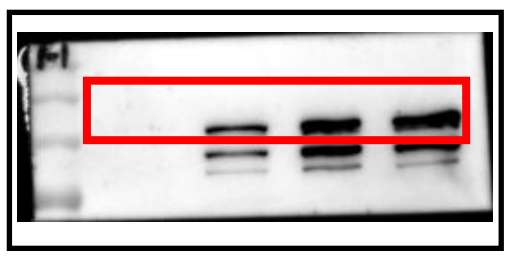

Fig. 2A β-actin

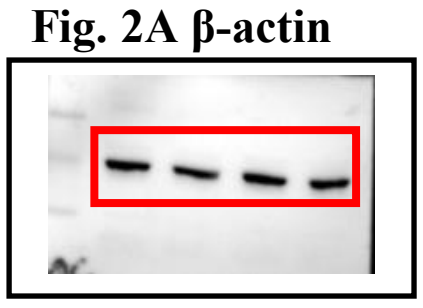

Fig. 2A PDCoV N

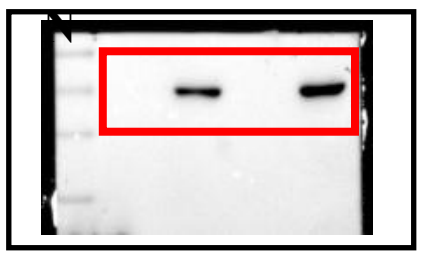

Fig. 2A DDX21

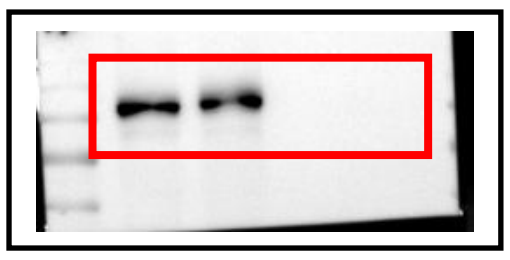

Fig. 2E β-actin

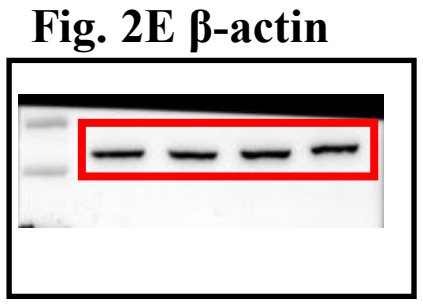

Fig. 2E PDCoV N

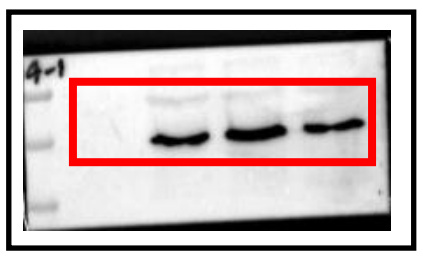

Fig. 2E DDX21

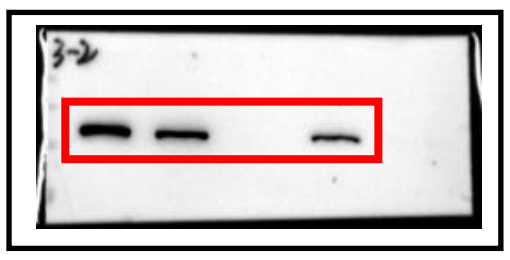

Fig. 2E Flag

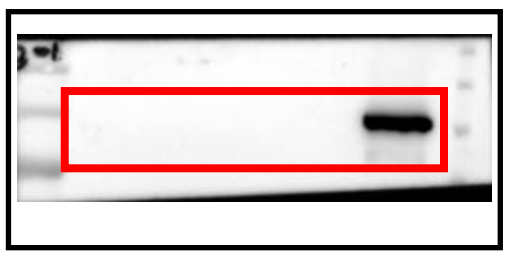

Fig. 3A β-actin

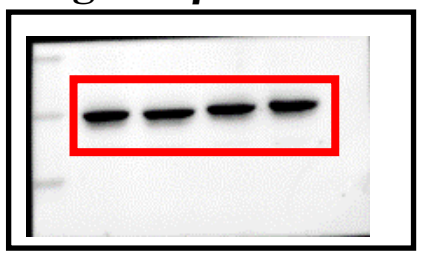

Fig. 3A PDCoV N

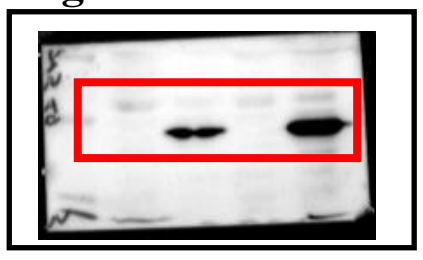

Fig. 3A DDX21

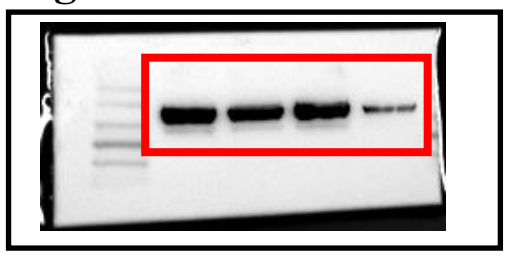

Fig. 3C β-actin

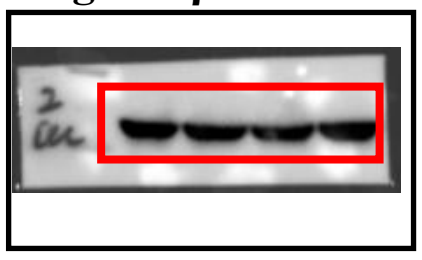

Fig. 3C PDCoV N

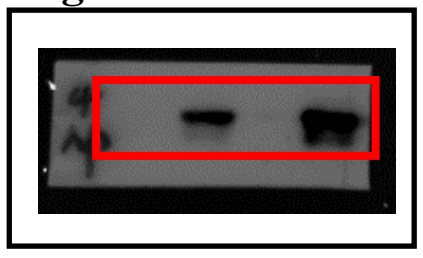

Fig. 3C DDX21

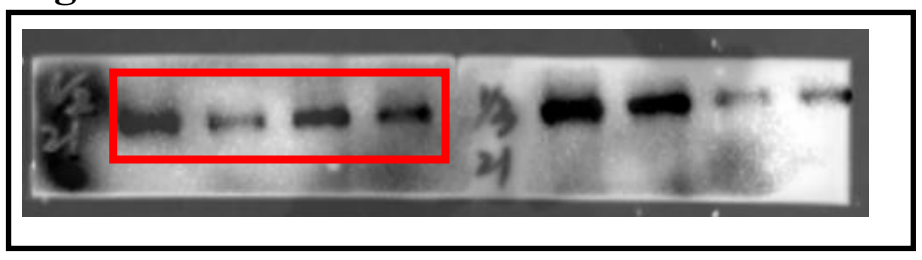

Fig. 3E β-actin

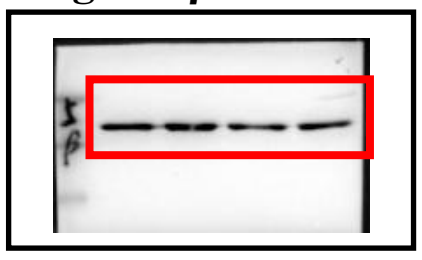

Fig. 3E PDCoV N

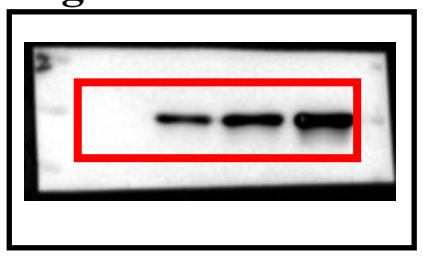

Fig. 3E DDX21

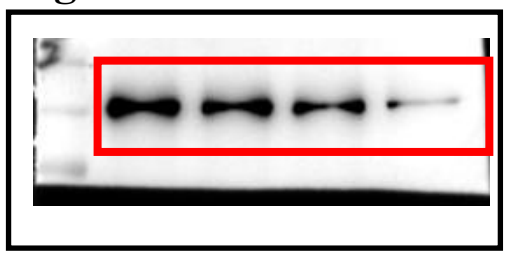

Fig. 3G β-actin

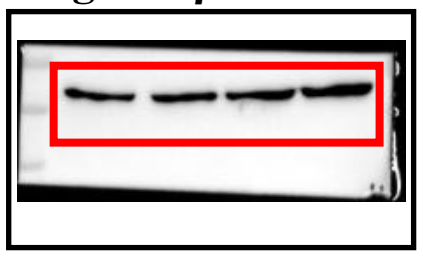

Fig. 3G PDCoV N

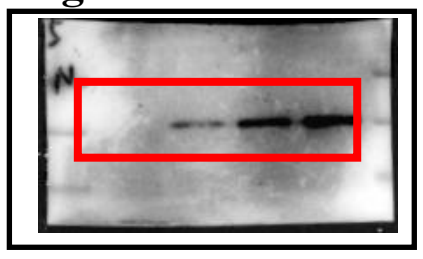

Fig. 3G DDX21

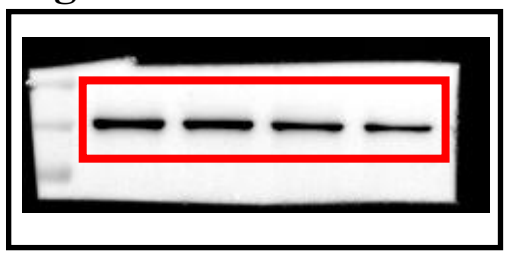

Fig. 4B β-actin

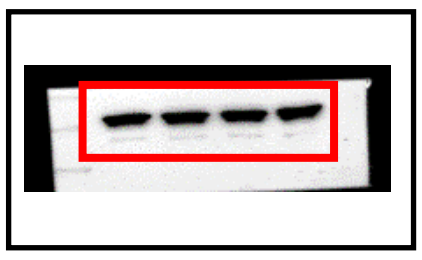

Fig. 4B Input HA

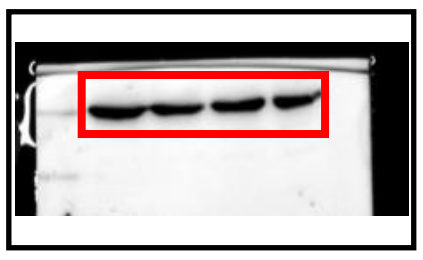

Fig. 4B Input Flag

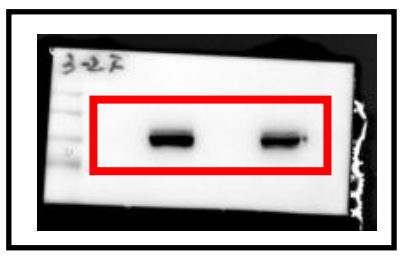

Fig. 4B IP HA

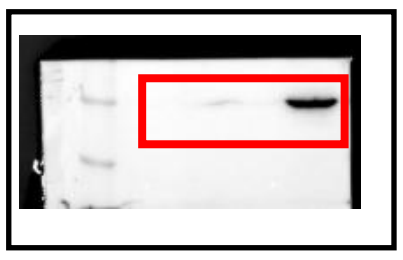

Fig. 4B IP Flag

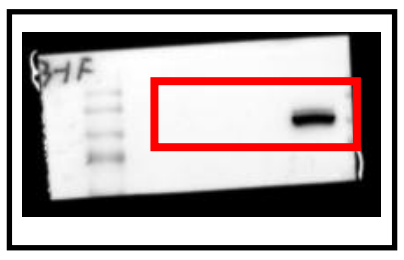

Fig. 4C β-actin

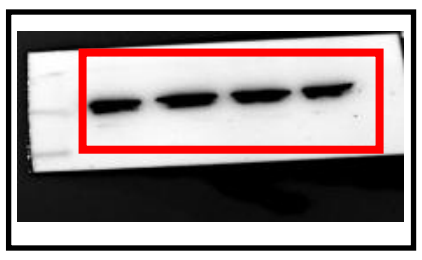

Fig. 4C Input HA

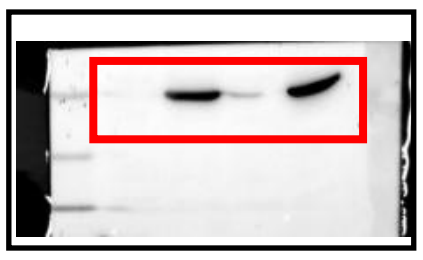

Fig. 4C Input Flag

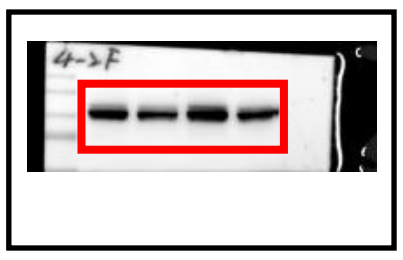

Fig. 4C IP HA

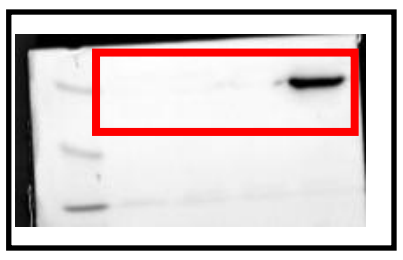

Fig. 4C IP Flag

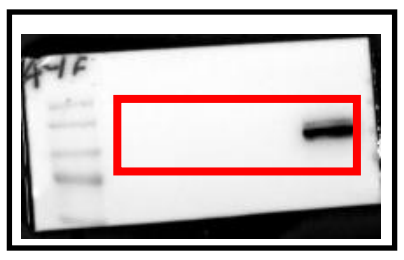

Fig. 4D β-actin

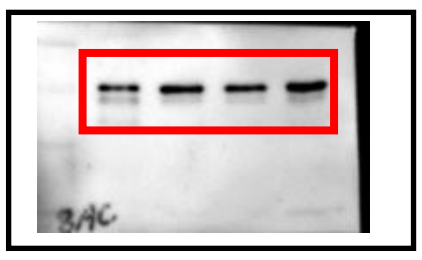

Fig. 4D Input DDX21

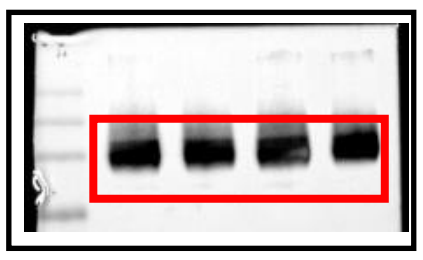

Fig. 4D Input PDCoV N

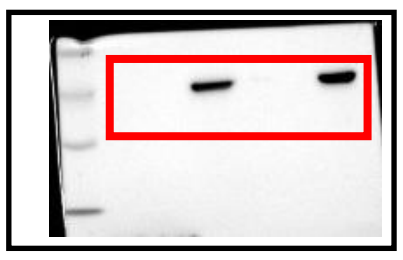

Fig. 4D IP DDX21

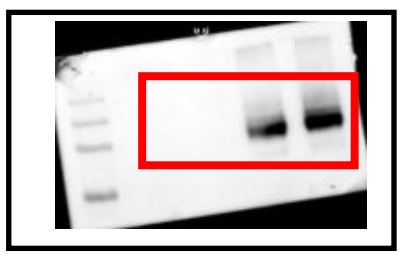

Fig. 4D IP PDCoV N

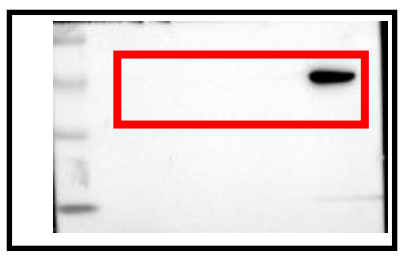

Fig. 4G β-actin

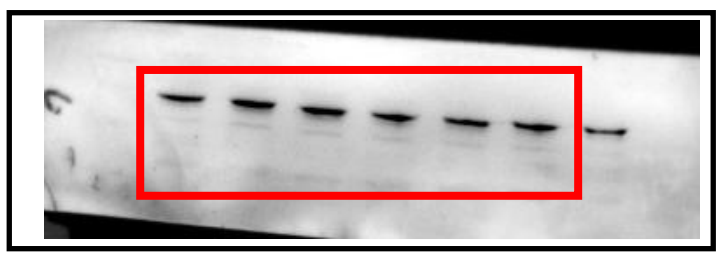

Fig. 4G Input HA

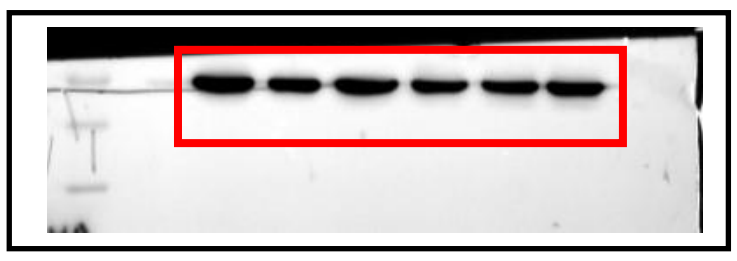

Fig. 4G Input Flag

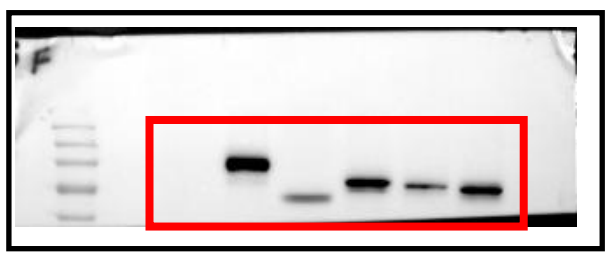

Fig. 4G IP HA

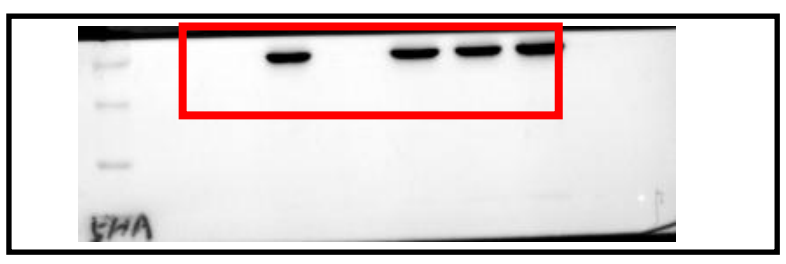

Fig. 4G IP Flag

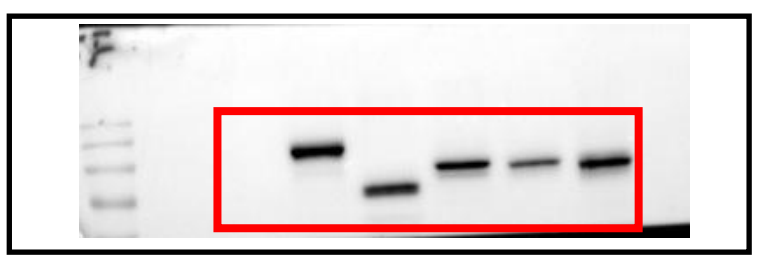

Fig. 4H β-actin

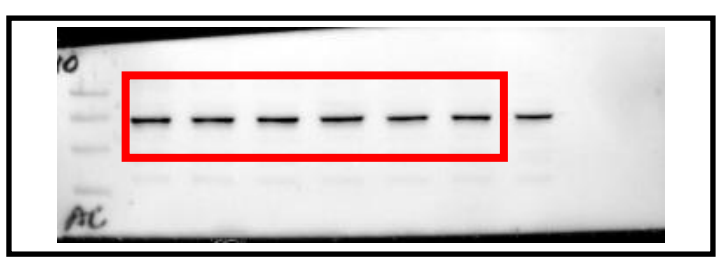

Fig. 4H Input HA

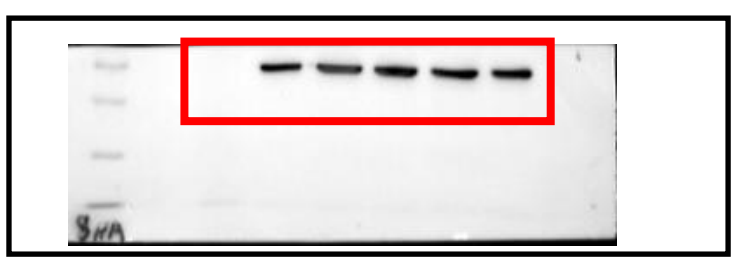

Fig. 4H Input Flag

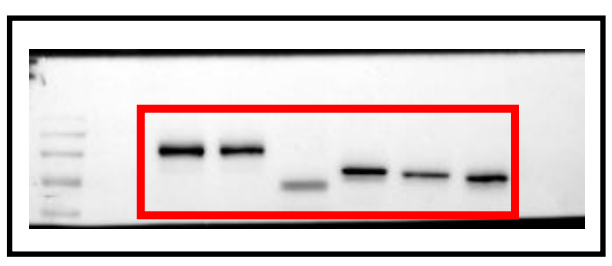

Fig. 4H IP HA

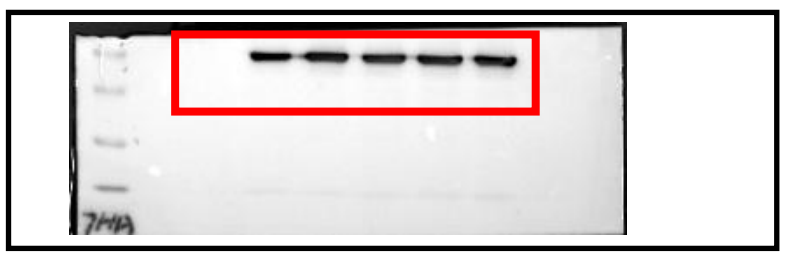

Fig. 4H IP Flag

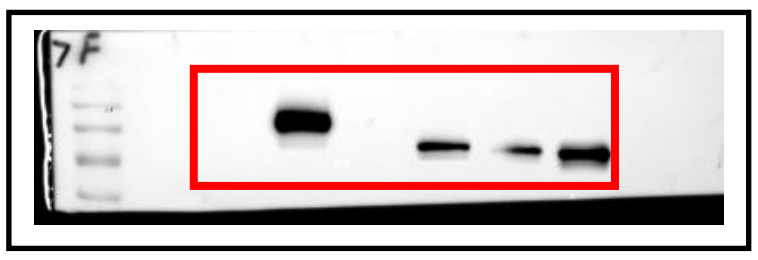

Fig. 5C β-actin

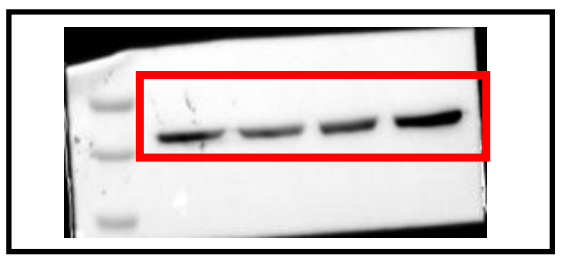

Fig. 5C Input HA

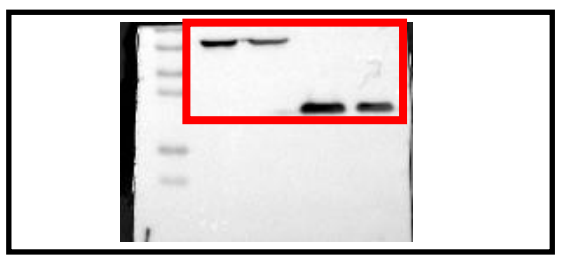

Fig. 5C Input Flag

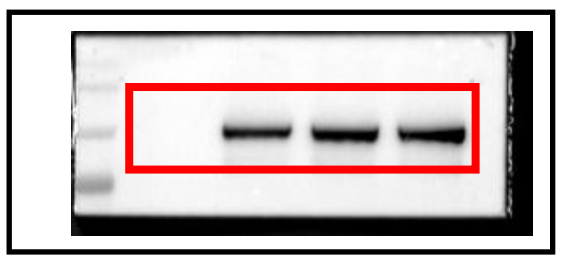

Fig. 5C IP HA

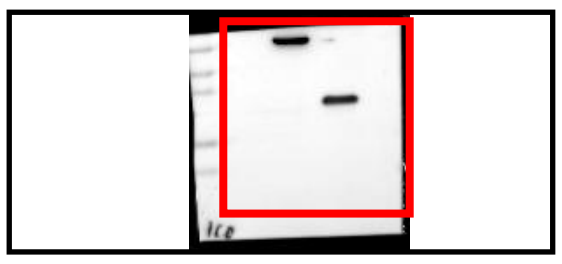

Fig. 5C IP Flag

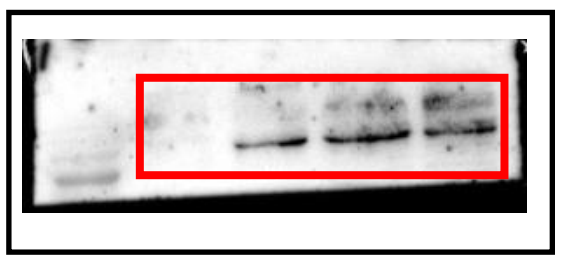

Fig. 5D β-actin

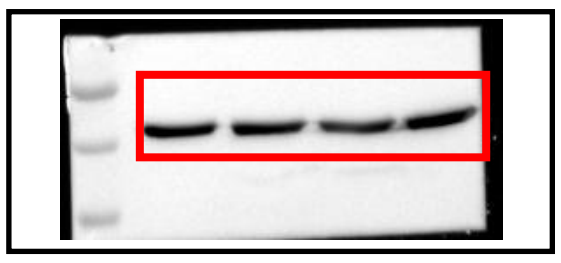

Fig. 5D Input HA

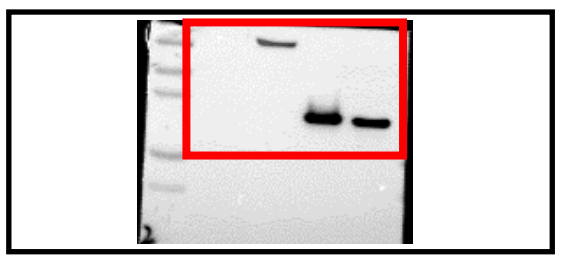

Fig. 5D Input Flag

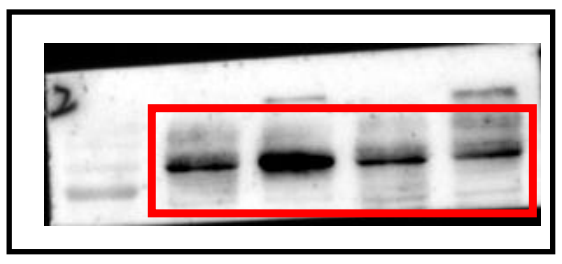

Fig. 5D IP HA

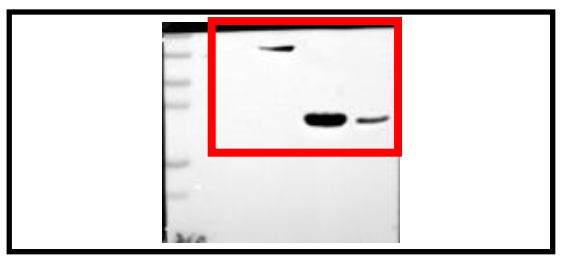

Fig. 5D IP Flag

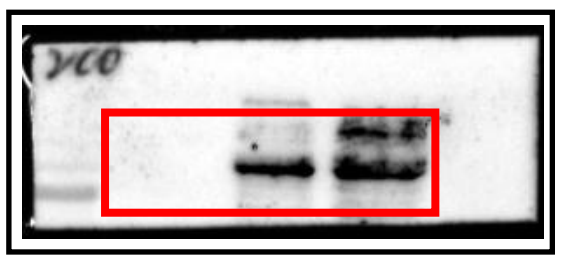

Fig. 6D β-actin

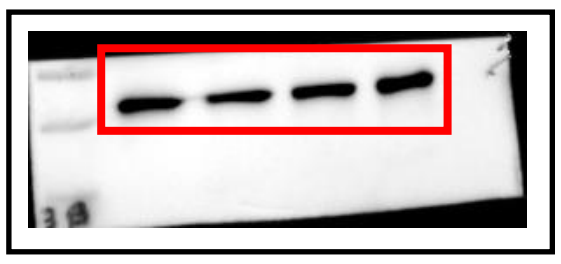

Fig. 6D PDCoV N

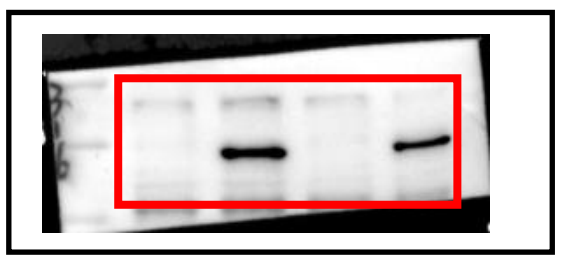

Fig. 6D Flag

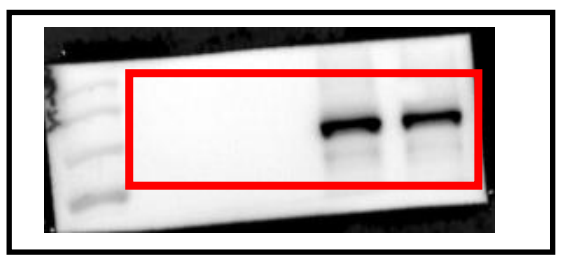

Fig. 6D IRF3

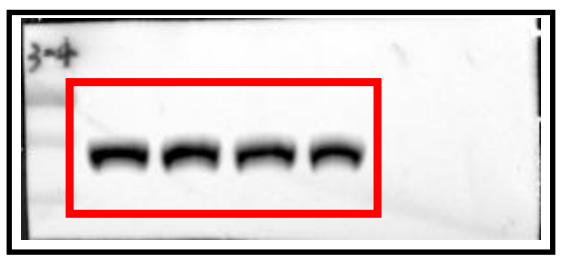

Fig. 6D pIRF3

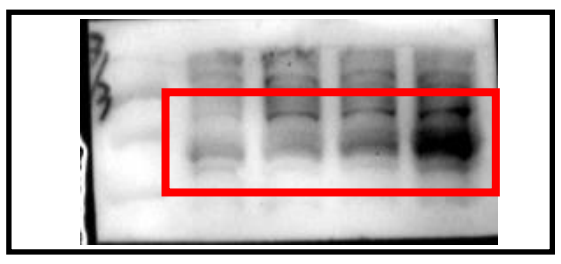

Fig. 6D TBK1

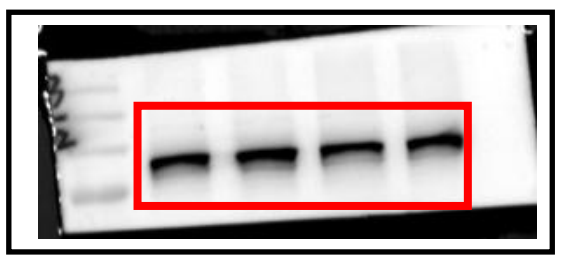

Fig. 6D pTBK1

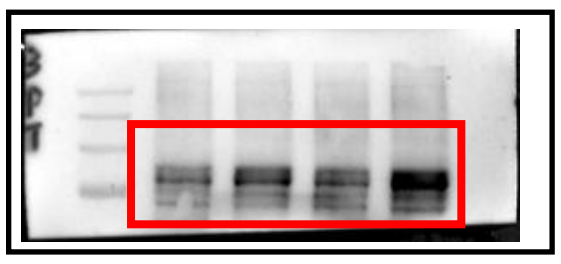

Fig. 6E β-actin

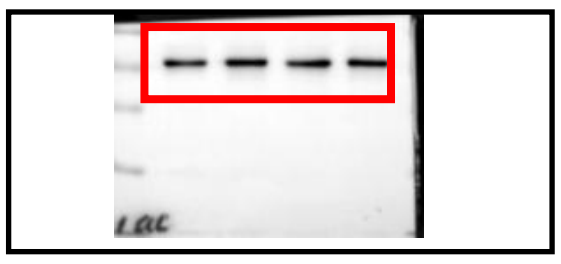

Fig. 6E PDCoV N

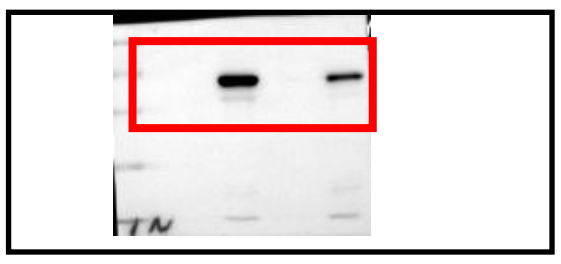

Fig. 6E Flag

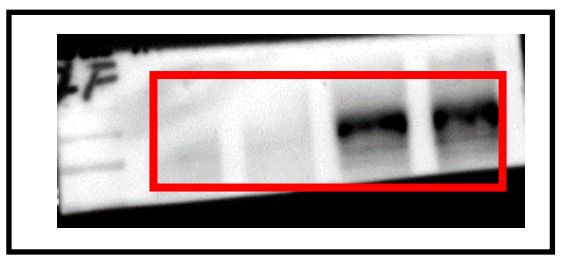

Fig. 6E IRF3

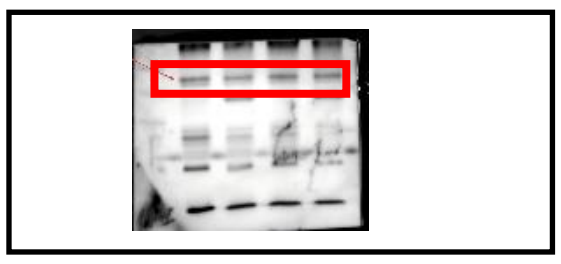

Fig. 6E pIRF3

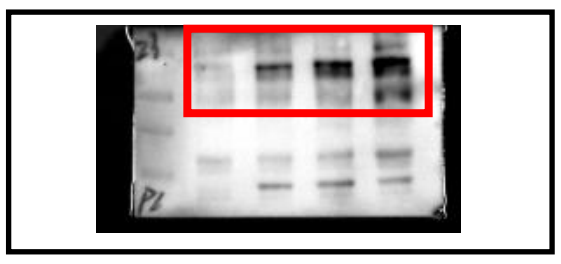

Fig. 6E TBK1

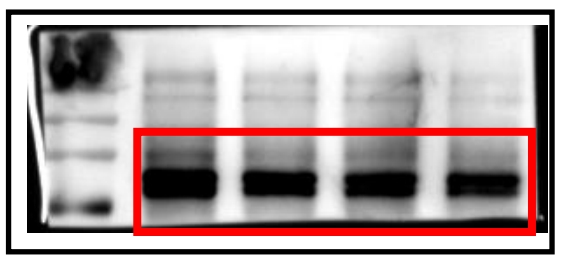

Fig. 6E pTBK1

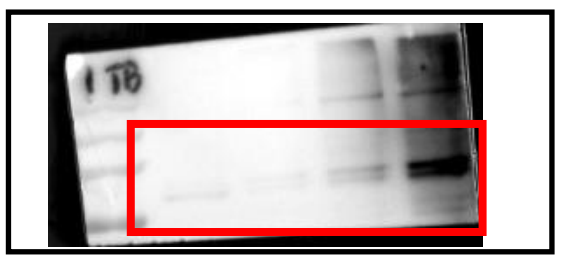

Fig. 6F β-actin

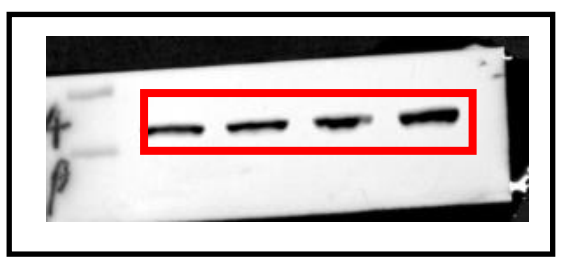

Fig. 6F PDCoV N

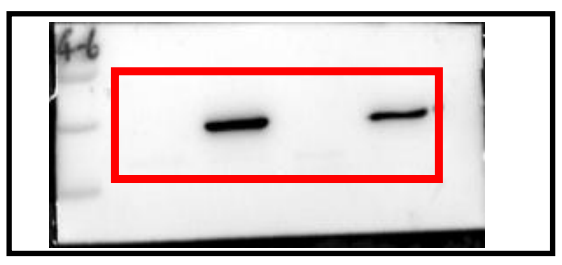

Fig. 6F Flag

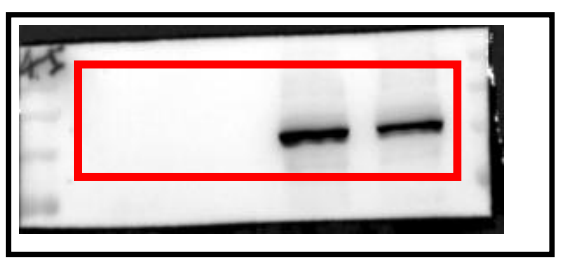

Fig. 6F IRF3

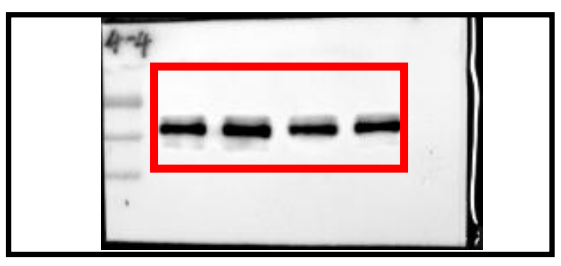

Fig. 6F pIRF3

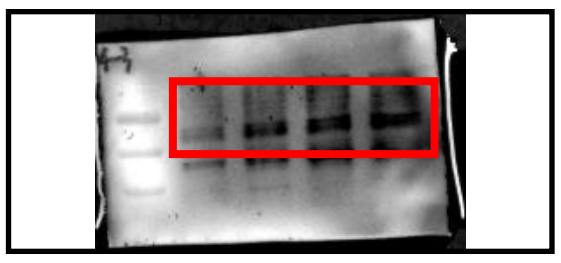

Fig. 6F TBK1

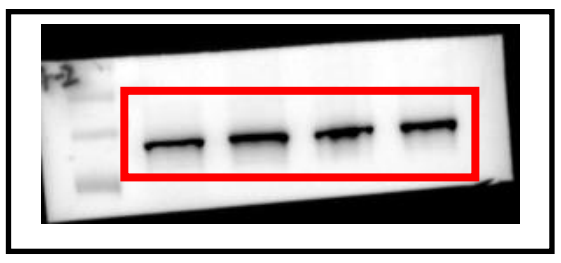

Fig. 6F pTBK1

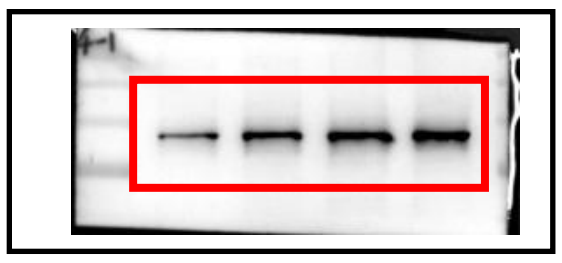

Fig. 6H β-actin

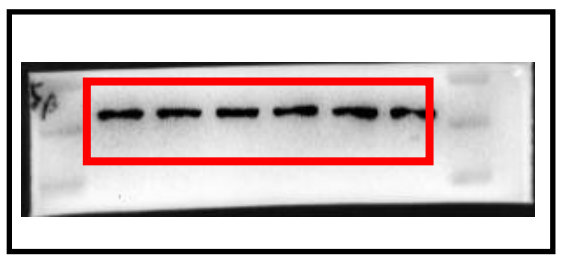

Fig. 6H PDCoV N

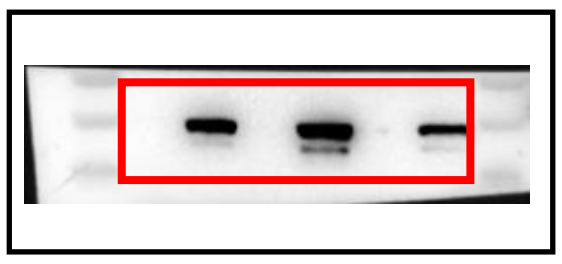

Fig. 6H DDX21

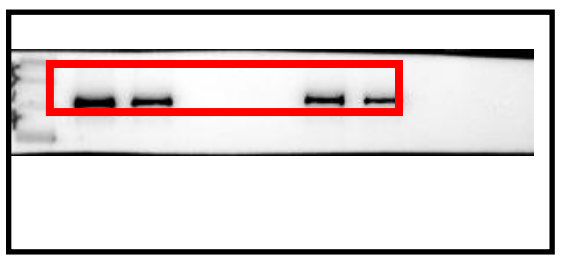

Fig. 6H Flag

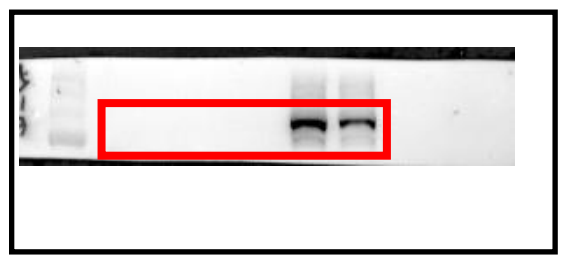

Fig. 6H IRF3

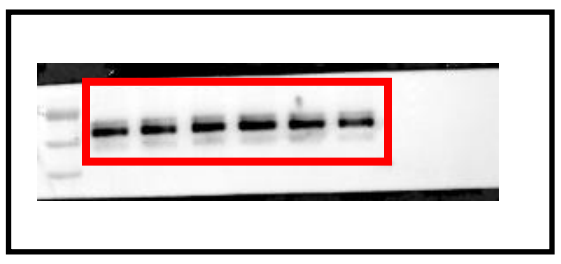

Fig. 6H pIRF3

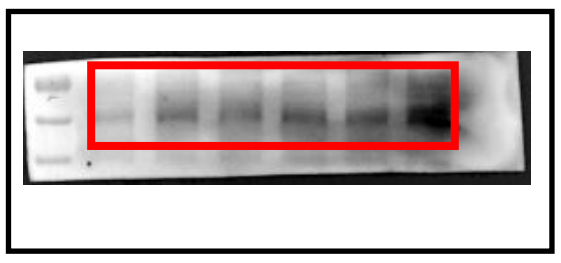

Fig. 6H TBK1

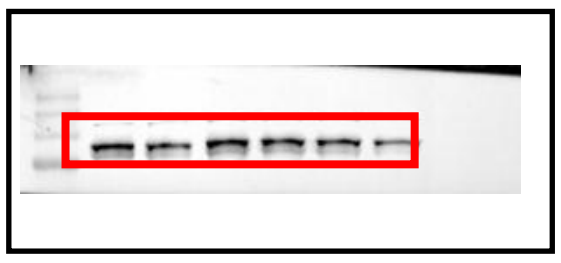

Fig. 6H pTBK1

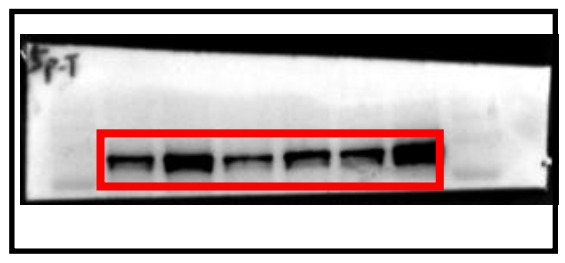

Fig. 6K β-actin

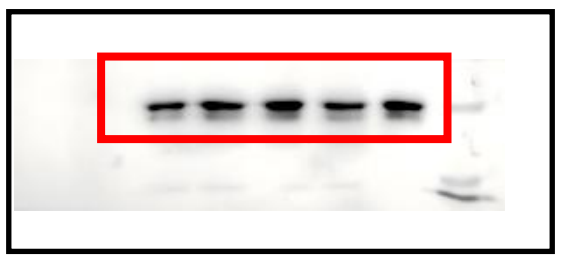

Fig. 6K PDCoV N

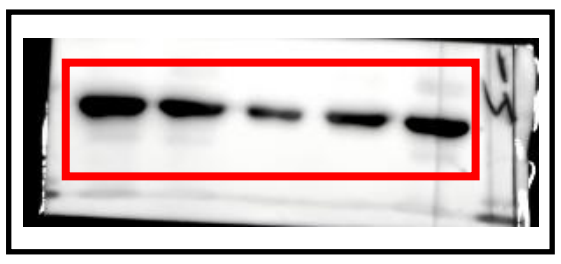

Fig. 6K Flag

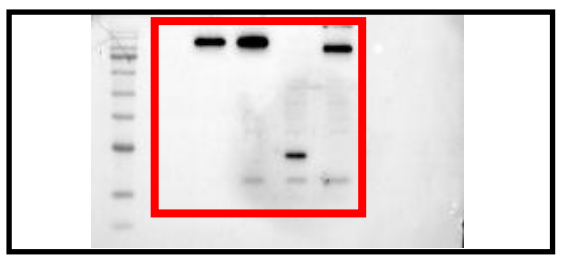

Fig. 6K IRF3

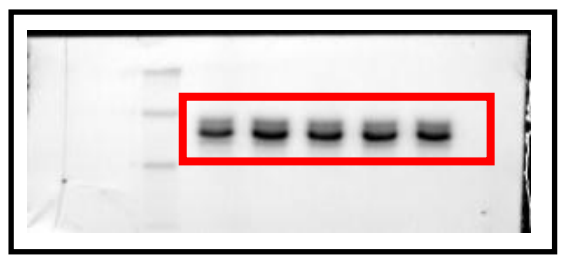

Fig. 6K pIRF3

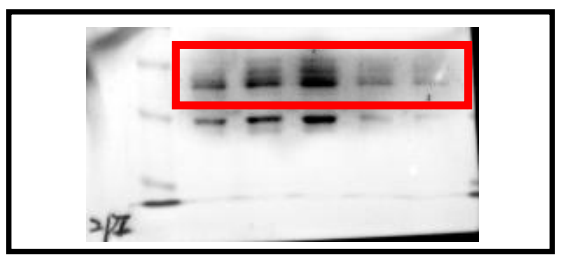

Fig. 6K TBK1

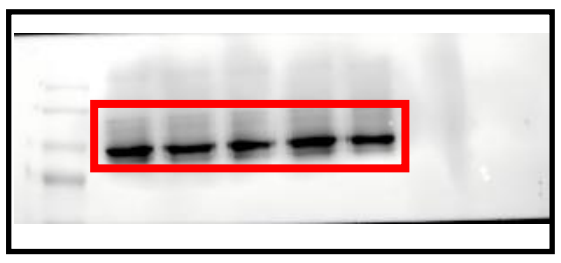

Fig. 6K pTBK1

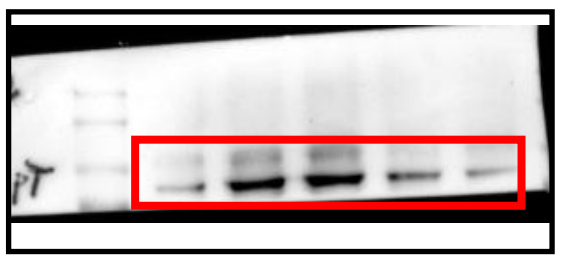

Fig. 7A β-actin

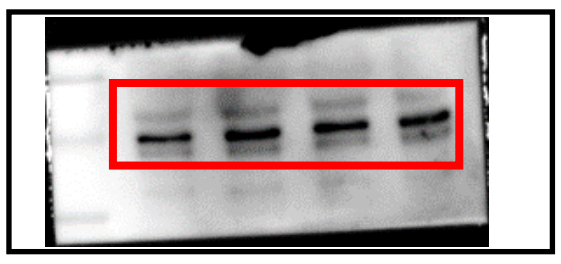

Fig. 7A IBV N

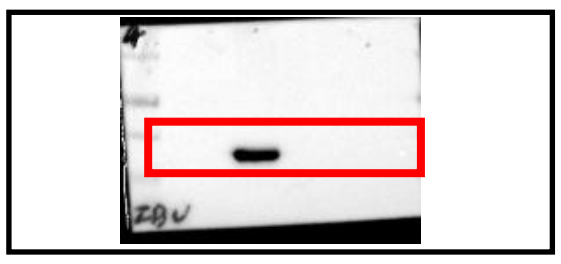

Fig. 7A PEDV N

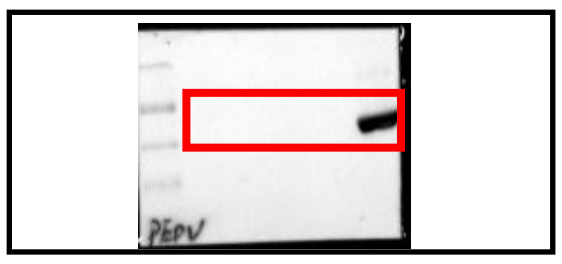

Fig. 7A DDX21

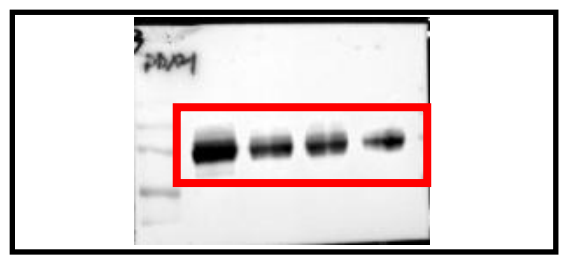

Fig. 7C β-actin

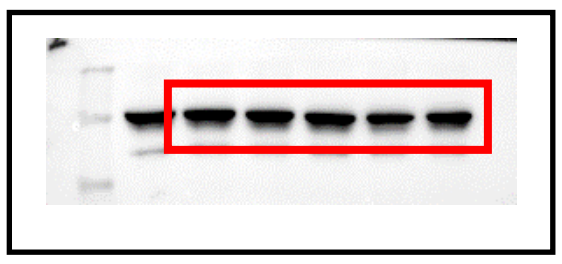

Fig. 7C input HA

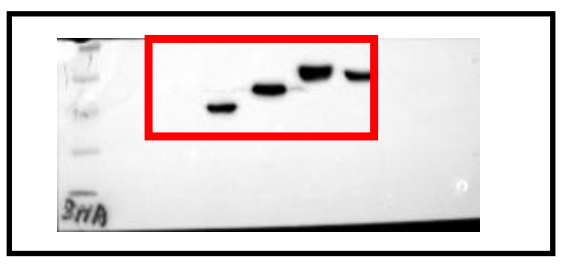

Fig. 7C input Flag

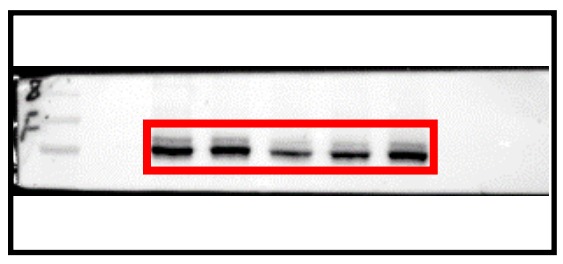

Fig. 7C IP HA

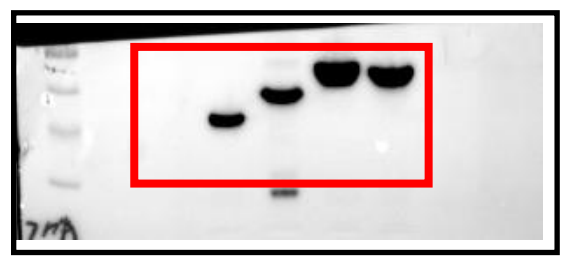

Fig. 7C IP Flag

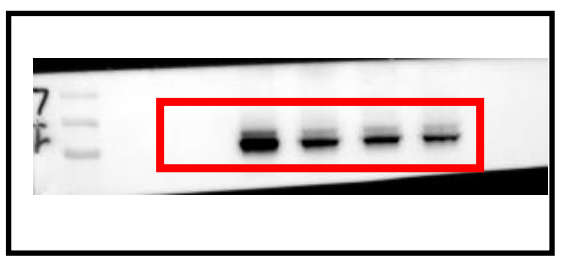

Fig. 7D β-actin

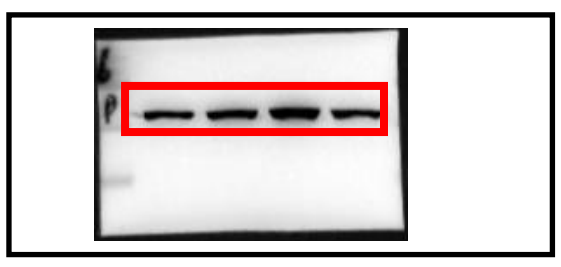

Fig. 7D IBV N

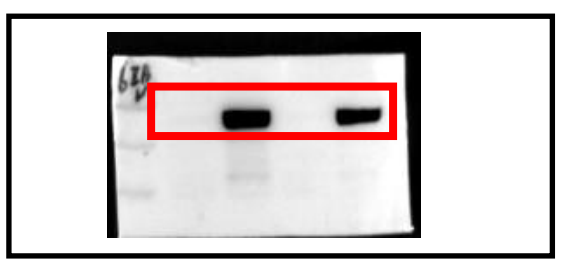

Fig. 7D Flag

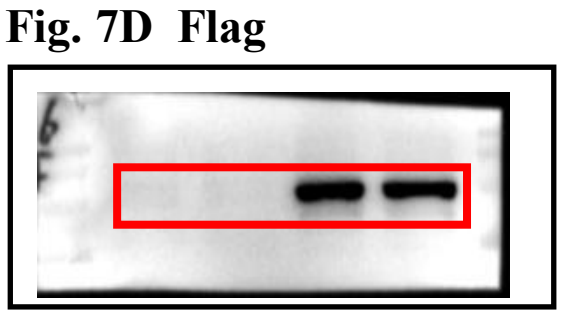

Fig. 7E β-actin

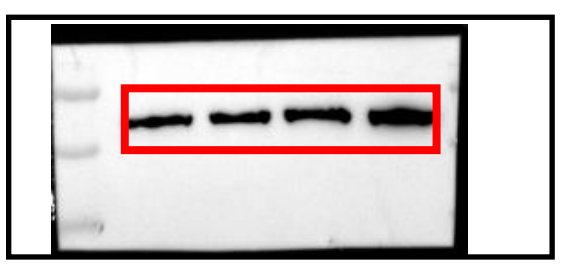

Fig. 7E PEDV N

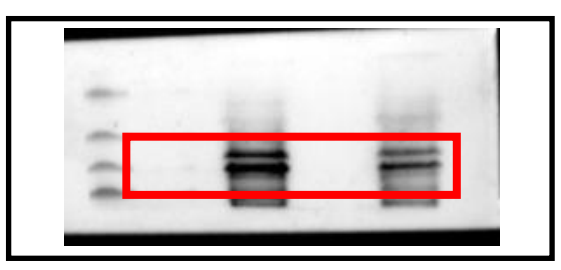

Fig. 7E Flag

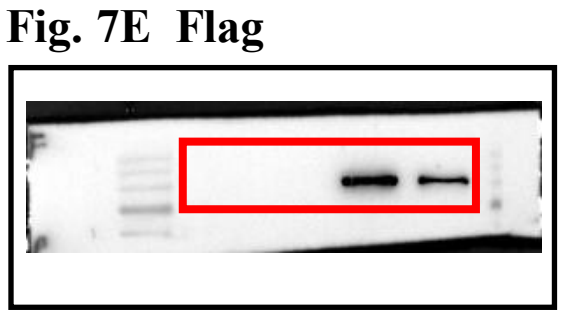

Fig.1A	12h Vector PDCoV			12h Flag-DDX21 PDCoV			24h Vector PDCoV			24h Flag-DDX21 PDCoV		
	0.9849901	1.011044201	1.0039657	0.7342575	0.6737254	0.6022026	1.2871362	1.2149410	1.3078394	0.9024115	0.8597983	0.8732213

Fig.1B	12h Vector PDCoV			12h Flag-DDX21 PDCoV			24h Vector PDCoV			24h Flag-DDX21 PDCoV		
	1.105306	0.986432	0.908262	0.577367	0.539338	0.600023	1.168832	1.188701	1.180783	0.811857	0.809723	0.772315

Fig.1C	12h Vector PDCoV			12h Flag-DDX21 PDCoV			24h Vector PDCoV			24h Flag-DDX21 PDCoV		
	0.908573	1.051160	1.040267	0.786545	0.807643	0.768964	1.452774	1.567432	1.495422	0.896754	1.098655	0.967543

Fig.1D	12h Vector PDCoV			12h Flag-DDX21 PDCoV			24h Vector PDCoV			24h Flag-DDX21 PDCoV		
	1.03	1.11	0.95	0.43	0.48	0.40	21.31	22.17	24.25	12.312	10.23	9.74

Fig.1E	12h Vector PDCoV			12h Flag-DDX21 PDCoV			24h Vector PDCoV			24h Flag-DDX21 PDCoV		
	0.98	1.11	0.91	0.63	0.58	0.60	19.87	20.61	18.98	10.88	11.12	11.56

Fig.1F	12h Vector PDCoV			12h Flag-DDX21 PDCoV			24h Vector PDCoV			24h Flag-DDX21 PDCoV		
	1.07	1.05	0.94	0.65	0.66	0.70	10.45	11.20	10.03	6.14	6.17	6.63

Fig.1G	12h Vector PDCoV			12h Flag-DDX21 PDCoV			24h Vector PDCoV			24h Flag-DDX21 PDCoV		
	7.105	6.619	6.541	5.875	5.875	5.884	7.737	8.100	8.167	6.780	6.568	6.375

Fig.1H	12h Vector PDCoV			12h Flag-DDX21 PDCoV			24h Vector PDCoV			24h Flag-DDX21 PDCoV		
	5.641	5.200	5.500	4.875	4.875	4.884	7.290	6.800	6.800	5.200	5.100	4.769

Fig.1I	12h Vector PDCoV			12h Flag-DDX21 PDCoV			24h Vector PDCoV			24h Flag-DDX21 PDCoV		
	5.700	5.429	5.333	4.200	3.771	4.194	5.900	6.231	6.200	5.000	4.882	4.800

Fig.1J	Vector PDCoV			Flag-DDX21 PDCoV		
	28.4	27.9	30.1	14.7	13.9	12.99

Fig.1K	Vector PDCoV			Flag-DDX21 PDCoV		
	26.4	24.9	29.1	17.3	17.0	16.7

Fig.1L	Vector PDCoV			Flag-DDX21 PDCoV		
	38.9	36.7	39.4	14.1	13.6	13.9

Fig.1M	Flag-DDX21 (0μg)PDCoV			Flag-DDX21 (0.5μg)PDCoV			Flag-DDX21 (1μg)PDCoV			Flag-DDX21 (1.5μg)PDCoV		
	1.077820	0.975438	0.946742	0.864918	0.875000	0.892191	0.761291	0.748681	0.674376	0.617741	0.601949	0.631580

Fig.1N	Flag-DDX21 (0μg)PDCoV			Flag-DDX21 (0.5μg)PDCoV			Flag-DDX21 (1μg)PDCoV			Flag-DDX21 (1.5μg)PDCoV		
	0.987554	1.104332	0.908114	0.728763	0.689643	0.712456	0.416484	0.457332	0.424567	0.391520	0.389643	0.367453

Fig.1 O	Flag-DDX21 (0μg)PDCoV			Flag-DDX21 (0.5μg)PDCoV			Flag-DDX21 (1μg)PDCoV			Flag-DDX21 (1.5μg)PDCoV		
	1.122312	0.986755	0.890933	0.649702	0.589543	0.612345	0.475111	0.425655	0.501233	0.027876	0.123344	0.134563

Fig.1P	Flag-DDX21 (0μg)PDCoV			Flag-DDX21 (0.5μg)PDCoV			Flag-DDX21 (1μg)PDCoV			Flag-DDX21 (1.5μg)PDCoV		
	1.07	0.95	0.94	0.83	0.77	0.79	0.54	0.55	0.59	0.43	0.47	0.44

Fig.1 Q	Flag-DDX21 (0μg)PDCoV			Flag-DDX21 (0.5μg)PDCoV			Flag-DDX21 (1μg)PDCoV			Flag-DDX21 (1.5μg)PDCoV		
	0.99	1.00	1.01	0.58	0.60	0.64	0.38	0.40	0.37	0.32	0.36	0.30

Fig.1R	Flag-DDX21 (0μg)PDCoV			Flag-DDX21 (0.5μg)PDCoV			Flag-DDX21 (1μg)PDCoV			Flag-DDX21 (1.5μg)PDCoV		
	0.96	1.08	0.96	0.36	0.44	0.49	0.27	0.28	0.32	0.07	0.07	0.06

Fig. 2B	WT PDCoV			DDX21$^{-/-}$ PDCoV		
	0.993210	0.906148	1.100642	1.804423	1.856438	2.535213

Fig. 2C	WT PDCoV			DDX21$^{-/-}$ PDCoV		
	0.98	1.03	0.99	1.66	1.69	1.71

Fig. 2D	WT PDCoV			DDX21$^{-/-}$ PDCoV		
	5.591	5.500	5.600	6.711	6.611	6.900

Fig. 2F	WT PDCoV			DDX21$^{-/-}$ PDCoV			DDX21$^{-/-}$+Flag-DDX21 PDCoV		
	0.987565	1.106532	0.905903	1.334456	1.452243	1.495321	1.140196	1.124445	1.196977

Fig. 2G	WT PDCoV			DDX21$^{-/-}$ PDCoV			DDX21$^{-/-}$+Flag-DDX21 PDCoV		
	0.93	1.03	1.04	1.74	1.88	1.81	1.23	0.98	1.12

Fig. 2H	WT PDCoV			DDX21$^{-/-}$ PDCoV			DDX21$^{-/-}$+Flag-DDX21 PDCoV		
	5.200	4.800	4.711	5.800	6.200	6.231	4.800	5.000	4.882

Fig.3B	12h Mock			12h PDCoV			24h Mock			24h PDCoV		
	0.976834	0.989845	1.033321	0.820951	0.819756	0.767432	0.937452	1.034521	1.028027	0.223353	0.316673	0.278421

Fig.3D	12h Mock			12h PDCoV			24h Mock			24h PDCoV		
	1.033216	0.879643	1.087141	0.652354	0.612345	0.438935	0.992453	0.816324	0.975432	0.417958	0.387122	0.351095

Fig.3F	PDCoV MOI=0			PDCoV MOI=0.1			PDCoV MOI=1			PDCoV MOI=5		
	1.017182	0.957886	1.024932	0.808317	0.785433	0.796532	0.561516	0.542134	0.551095	0.225774	0.253321	0.274232

Fig.3H	PDCoV MOI=0			PDCoV MOI=0.1			PDCoV MOI=1			PDCoV MOI=5		
	1.097703	0.992243	0.910054	0.823455	0.813456	0.767643	0.746477	0.684564	0.678322	0.423181	0.356678	0.389534

Fig.3I	12h Mock			12h PDCoV			24h Mock			24h PDCoV		
	1.03	1.10	0.94	0.65	0.59	0.61	1.04	0.99	1.02	0.14	0.16	0.20

Fig.3J	12h Mock			12h PDCoV			24h Mock			24h PDCoV		
	1.01	1.03	0.99	0.59	0.47	0.51	1.01	0.99	1.03	0.21	0.23	0.24

Fig.3K	PDCoV MOI=0			PDCoV MOI=0.1			PDCoV MOI=1			PDCoV MOI=5		
	1.00	1.04	0.96	0.71	0.74	0.79	0.69	0.64	0.7.0	0.36	0.32	0.34

Fig.3L	PDCoV MOI=0			PDCoV MOI=0.1			PDCoV MOI=1			PDCoV MOI=5		
	1.00	1.02	0.98	0.76	0.77	0.78	0.72	0.70	0.68	0.35	0.34	0.31

Fig.6A	Vector Mock			Vector PDCoV			Flag-DDX21 Mock			Flag-DDX21 PDCoV		
	IFN β											
	0.9481	1.0932	0.9648	27.5192	26.2740	25.4019	1.5535	1.1201	0.9031	34.5401	31.9800	31.6998
	OAS1											
	0.948136	1.093207	0.964776	3.169086	3.152435	3.026534	1.068411	1.125266	1.000670	8.473747	9.649138	7.304067
	ISG15											
	0.948136	1.093207	0.964776	3.182881	3.501889	3.34066	0.999122	1.087363	0.903899	5.802066	6.295263	5.329007
	ISG54											
	0.948136	1.093207	0.964776	6.234325	6.724530	6.868206	1.129966	1.229542	1.339647	8.489032	9.685322	9.925226

Fig.6B	Vector Mock			Vector PDCoV			Flag-DDX21 Mock			Flag-DDX21 PDCoV		
	IFN β											
	0.95	1.04	1.01	7.39	8.06	7.93	0.89	0.99	1.39	11.47	10.88	12.85
	OAS1											
	1.04	1.04	0.92	2.13	2.25	2.35	1.17	1.15	1.09	4.09	4.89	5.39
	ISG15											
	1.01	1.00	0.99	3.32	3.17	3.35	1.04	0.99	1.24	6.41	6.39	6.62
	ISG54											
	0.98	1.04	0.98	2.92	2.88	2.35	1.18	1.29	1.2	5.28	5.33	5.41

Fig.6C	Vector Mock			Vector PDCoV			Flag-DDX21 Mock			Flag-DDX21 PDCoV		
	IFN β											
	1.039016	0.943627	1.019946	5.092242	4.826579	6.106311	1.396092	1.036194	1.082370	9.148062	8.805264	7.435172
	OAS1											
	1.039016	0.943627	1.019946	2.726659	3.714671	2.791488	1.581756	1.207336	1.144032	8.107738	6.675147	9.047377
	ISG15											
	1.039016	0.943627	1.019946	4.252961	3.509262	2.929121	1.339375	0.994098	1.038399	7.128670	5.200077	6.655435
	ISG54											
	1.039016	0.943627	1.019946	4.640567	4.640567	6.107581	1.444023	1.112645	0.976501	9.913993	7.776205	7.195855

Fig.6 G	WT Mock			WT PDCoV			DDX21$^{-/-}$ Mock			DDX21$^{-/-}$ PDCoV		
	IFN β											
	1.08	1.00	1.16	4.12	4.30	4.00	1.01	1.02	0.99	2.26	2.2	2.19
	OAS1											
	1.07	0.94	0.99	3.17	3.21	3.19	1.04	1.06	0.90	1.85	1.74	1.80
	ISG15											
	1.04	1.06	0.97	2.22	2.30	2.20	1.02	1.07	0.99	1.01	1.06	1.03
	ISG54											
	1.00	1.07	0.93	7.6	7.74	7.46	1.07	1.11	1.09	1.29	1.26	1.27

Fig.6I	WT PDCoV			DDX21$^{-/-}$ PDCoV			DDX21$^{-/-}$ PDCoV+Flag-DDX21		
	IFN β								
	0.88	0.97	1.17	0.47	0.36	0.42	1.21	1.08	1.05
	OAS1								
	0.89	0.99	1.14	0.13	0.18	0.19	1.13	1.23	1.16
	ISG15								
	0.90	0.95	1.14	0.54	0.57	0.56	0.89	1.01	1.03
	ISG54								
	0.99	1.10	0.89	0.17	0.21	0.19	1.10	1.15	0.98

Fig. 6L	Vector PDCoV			Flag-DDX21(WT) PDCoV			Flag-DDX21(D126A) PDCoV			Flag-DDX21(1-125) PDCoV			Flag-DDX21(127-784) PDCoV		
	IFN β														
	0.95	0.97	1.09	7.08	6.60	5.34	21.39	19.44	21.15	1.44	1.46	1.72	0.81	0.76	0.72
	OAS1														
	1.01	1.16	0.85	7.95	8.42	9.57	80.00	80.72	96.84	1.65	1.56	1.62	0.64	0.45	0.45
	ISG15														
	0.94	1.09	0.98	9.57	9.54	9.42	60.28	66.32	61.55	1.24	1.22	1.19	0.33	0.42	0.36
	ISG54														
	1.26	0.87	0.92	9.07	8.13	8.92	52.64	55.11	57.82	1.56	1.49	1.54	0.84	0.87	0.79

Fig.7B	H1299 Mock			H1299 IBV			PK15 Mock			PK15 PEDV		
	0.997865	1.023132	0.979002	0.341999	0.453321	0.378542	1.088542	0.975563	0.935895	0.524345	0.612455	0.574245

Fig .7F	Vector IBV			Flag-DDX21 IBV			Vector PEDV			Flag-DDX21 PEDV			Vector MHV			Flag-DDX21 MHV		
	1.31	1.04	0.89	0.21	0.34	0.25	1.17	1.01	0.94	0.57	0.49	0.61	1.23	0.97	0.89	0.39	0.47	0.35

Fig.7 G	Vector Mock			Vector IBV			Flag-DDX21 Mock			Flag-DDX21 IBV		
	**IFN β**											
	1.023587	0.95663	1.021248	6.079559	6.929574	6.000849	1.00368	1.354725	1.194826	7.995196	8.58509	9.911706
	**OAS1**											
	1.023587	0.95663	1.021248	2.401607	2.200452	2.164149	0.921869	0.966829	1.049814	5.323838	6.12694	7.581413
	**ISG15**											
	1.023587	0.95663	1.021248	2.49793	2.161501	2.12938	0.922529	0.894322	1.008399	4.566535	4.440124	4.782285
	**ISG54**											
	1.023587	0.95663	1.021248	2.542063	2.421498	2.586677	1.096952	0.988628	0.922103	5.954031	5.496704	5.412393

Fig.7 H	Vector Mock			Vector PEDV			Flag-DDX21 Mock			Flag-DDX21 PEDV		
	**IFN β**											
	0.896722	0.900709	1.238105	3.812225	3.742321	3.159362	1.365157	1.425894	1.343472	6.466418	7.266027	7.656933
	**OAS1**											
	0.896722	0.900709	1.238105	2.416636	2.126528	1.925723	0.997116	1.078057	1.06469	4.220557	3.910989	4.311331
	**ISG15**											
	0.896722	0.900709	1.238105	1.616380	1.923810	1.931560	0.892918	1.271032	1.241858	3.345681	3.679985	3.372691
	**ISG54**											
	0.896722	0.900709	1.238105	2.386892	2.345074	2.520017	1.222612	1.204443	1.002336	4.864416	5.575753	4.806433

Fig.7I	Vector Mock			Vector MHV			Flag-DDX21 Mock			Flag-DDX21 MHV		
	**IFN β**											
	0.949035	1.007887	1.045457	2.637187	2.758913	2.681053	0.952726	0.874562	1.215065	5.268201	6.604322	6.666874
	**OAS1**											
	0.949035	1.007887	1.045457	12.544650	11.424820	12.204810	1.281500	1.059904	1.069943	27.781180	28.972920	29.114480
	**ISG15**											
	0.949035	1.007887	1.045457	4.819778	6.246148	5.975944	1.922833	1.205251	2.433332	7.135428	10.04357	8.826858
	**ISG54**											
	0.949035	1.007887	1.045457	3.671832	3.136814	5.581682	1.924211	1.308457	1.543031	10.714060	8.281163	8.583900
