# Supplementary material for: DEAD-box RNA helicase 21 interacts with the nucleocapsid protein and inhibits porcine deltacoronavirus infection through upregulation of interferon responses
Source: J Virol. 2026 Sep 30;100(10):e01169-26. doi: 10.1128/jvi.01169-26 (PMC13712866; doi:10.1128/jvi.01169-26)
Supplement: Supplementary file 2 [file jvi.01169-26-s0002.docx]

**Supplementary Table S1. Primer sequences used in this study**

| **Gene ID** | **Sequence (5′→3′)** |
| --- | --- |
| Pig-DDX21(1-784)F | CCGTCAGAATTAAGCTTatgccggggaaacttcgta |
| Pig-DDX21(1-784)R | AGTCAGCCCGGGATCCctgtccaaacgctttgct |
| Pig-DDX21(218-784)F | GTCAGAATTAAGCTTtttcaccatgtctatagcgg |
| Pig-DDX21(218-784)R | AAGCTTAATTCTGACGGTTC |
| Pig-DDX21(1-217+398-784)F | atacaagcaaagacggtggacctgattggtaagaaga |
| Pig-DDX21(1-217+398-784)R | cgtctttgcttgtatgggaa |
| Pig-DDX21(1-397+582-784)F | atctacatatgaacagaggcttttggattctgtgcc |
| Pig-DDX21(1-397+582-784)R | ctgttcatatgtagatttcatgtatttctt |
| Pig-DDX21(1-581)F | agtaaagatgccatcGGATCCCGGGCTGACTACAA |
| Pig-DDX21(1-581)R | gatggcatctttactagaagct |
| qPCR-pig-DDX21F | cggacaggaactgggaagac |
| qPCR-pig-DDX21R | cgtgatgtcactgaagtctc |
| qPCR-human-DDX21F | aaagaagcccaggagctgtc |
| qPCR-human-DDX21R | cagggatgtctaacccacgt |
| qPCR-pig-IFN-βF | ACCACAGCTCTTTCCATGAG |
| qPCR-pig-IFN-βR | CAGGGACCTCAAAGTTCATCC |
| qPCR-pig-ISG15F | cctgttgatggtgcaaagct |
| qPCR-pig-ISG15R | tgcacataggcttgaggtca |
| qPCR-pig-ISG54F | ctggcaaagagccctaagga |
| qPCR-pig-ISG54R | ctcagagggtcaatggaattcc |
| qPCR-pig-OAS1F | aagcataagaagctttgcatctt |
| qPCR-pig-OAS1R | aagcataagaagctttgcatctt |
| qPCR-human-IFN-βF | ACCTACAGGGCGGACTTCAA |
| qPCR-human-IFN-βR | ACACTGTCTGCTGGTGGAGT |
| qPCR-human-ISG15F | acctgacggtgaagatgctg |
| qPCR-human-ISG15R | gcatttgtccaccaccagc |
| qPCR-human-ISG54F | attgaccctctgaggcaagc |
| qPCR-human-ISG54R | ggtggatggccttgtcttca |
| qPCR-human-OAS1F | cctaccctgtgtgtgtgtcc |
| qPCR-human-OAS1R | tggacctcaaacttcacgga |
| qPCR-PDCoV-NF | CCCAGCTCAAGGTTTCAGAG |
| qPCR-PDCoV-NR | ATTGGCACCAGTACGAGACC |
| qPCR-IBV-NF | GACGGAGGACCTGATGGTAA |
| qPCR-IBV-NR | CCCTTCTTCTGCTGATCCTG |
| qPCR-PEDV-NF | ATGCCCAACAACGACACCT |
| qPCR-PEDV-NR | TGTAGCCAACGCCAGAACAC |
| qPCR-MHV-NF | TGGAAGGTCTGCACCTGCTA |
| qPCR-MHV-NR | TTTGGCCCACGGGATTG |
| qPCR-pig-β-actinF | tccctggagaagagctacga |
| qPCR-pig-β-actinR | tagaggtccttgcggatgtc |
| qPCR-human-β-actinF | GATCTGGCACCACACCTTCT |
| qPCR-human-β-actinR | GGGGTGTTGAAGGTCTCAAA |

**Supplementary Table S2. Amino acid identity of DDX21 among different species**

| Porcine | Human | Ferret | Chimpanzee | Chicken | Bovine | Murine |  |
| --- | --- | --- | --- | --- | --- | --- | --- |
| *** | 89.8% | 91.8% | 90% | 66.9% | 93.2% | 85.5% | Porcine |
|  | *** | 92.1% | 99.9% | 67% | 91.1% | 86.5% | Human |
|  |  | *** | 91.9% | 67.3% | 92.4% | 86.1% | Ferret |
|  |  |  | *** | 66.9% | 91.1% | 86.6% | Chimpanzee |
|  |  |  |  | *** | 66.1% | 66.1% | Chicken |
|  |  |  |  |  | *** | 84.8% | Bovine |
|  |  |  |  |  |  | *** | Murine |
